# Author Correction: Improved machine learning algorithm for predicting ground state properties

**DOI:** 10.1038/s41467-024-46164-4

**Published:** 2024-02-26

**Authors:** Laura Lewis, Hsin-Yuan Huang, Viet T. Tran, Sebastian Lehner, Richard Kueng, John Preskill

**Affiliations:** 1 California Institute of Technology, Pasadena, CA USA; 2 Massachusetts Institute of Technology, Cambridge, MA USA; 3 Johannes Kepler University, Linz, Austria; 4 AWS Center for Quantum Computing, Pasadena, CA USA; 5 Present Address: University of Cambridge, Cambridge, UK; 6 Present Address: Google Quantum AI, Venice, CA USA

**Keywords:** Quantum information, Computer science, Quantum mechanics

Correction to: *Nature Communications* 10.1038/s41467-024-45014-7, published online 30 January 2024

The original version of this Article incorrectly acknowledged Laura Lewis as a corresponding author instead of Hsin-Yuan Huang. This has now been corrected in both the PDF and HTML versions of the Article.

