# Supplementary material for: Improved machine learning algorithm for predicting ground state properties
Source: Nat Commun. 2024 Jan 30;15:895. doi: 10.1038/s41467-024-45014-7 (PMC10828424; doi:10.1038/s41467-024-45014-7)
Supplement: Supplementary file 1 — Supplementary Information [file 41467_2024_45014_MOESM1_ESM.pdf]

Improved machine learning algorithm for predicting ground state properties

Laura Lewis,[1,*] Hsin-Yuan Huang,[1] Viet T. Tran,[2] Sebastian Lehner,[2] Richard Kueng,[2] and John Preskill[1,3]

[1] *California Institute of Technology, Pasadena, CA, USA*
[2] *Johannes Kepler University, Linz, Austria*
[3] *AWS Center for Quantum Computing, Pasadena, CA, USA*

(Dated: December 7, 2023)

Supplementary Information

Contents

I. Simple form for ground state property 2
A. Approximation by a sum of smooth functions 2
B. Simplification using discretization 10
C. Simple form for ground state property 12
D. Technical lemmas for finding constants and bounding integrals 13

II. Norm inequality for observables 18
A. Facts and lemmas 18
B. Proof of Theorem 4 19

III. ML algorithm and sample complexity 23
A. ML algorithm 23
B. Rigorous guarantee 25
C. ℓ_1-Norm bound on coefficients of linear hypothesis 26
D. Training error bound 28
E. Prediction error bound 29
F. Computational time for training and prediction 30

IV. Details of numerical experiments 30

These appendices provide detailed proofs of the statements in the main text. We discuss our main contribution that $\mathrm{Tr}(O\rho)$ can be approximated by a machine learning model given training data scaling logarithmically in system size, where O is an unknown observable and ρ is the ground state of a Hamiltonian. The proof of this result has three main parts. The first two parts yield important results necessary for the design of the ML algorithm and its sample complexity.

We recommend that readers start with Section I, which derives a simpler form for the ground state property $\mathrm{Tr}(O\rho(x))$ that we wish to predict. In Section II, we give a norm inequality characterizing the Pauli coefficients of any observable that can be written as a sum of geometrically local observables. The norm inequality reveals a structure of the ground state property $\mathrm{Tr}(O\rho(x))$ that we can use to design an ML algorithm that uses very few training data. In Section III, we present our ML algorithm and prove its sample complexity using standard tools in ML theory, including known guarantees on the LASSO (least absolute shrinkage and selection operator) algorithm's performance. Finally, in Section IV, we describe numerical experiments performed to assess the performance of the algorithm in practice.

*Electronic address: llewis@alumni.caltech.edu

I. SIMPLE FORM FOR GROUND STATE PROPERTY

This section is dedicated to deriving a simpler form for the ground state property $\mathrm{Tr}(O\rho(x))$ as a function of x. Note that throughout this section, log denotes the natural logarithm. We consider the assumptions (a)-(d) from Appendix F.5 of [1], with (b) and (d) adjusted for our setting, which we reproduce here for convenience:

(a) *Physical system:* We consider n finite-dimensional quantum systems that are arranged at locations, or sites, in a d-dimensional space, e.g., a spin chain ($d = 1$), a square lattice ($d = 2$), or a cubic lattice ($d = 3$). Unless specified otherwise, our big-$\mathcal{O}, \Omega, \Theta$ notation is with respect to the thermodynamic limit $n \to \infty$.

(b) *Hamiltonian:* $H(x)$ decomposes into a sum of geometrically local terms $H(x) = \sum_{j=1}^{L} h_j(\vec{x}_j)$, each of which only acts on an $\mathcal{O}(1)$ number of sites in a ball of $\mathcal{O}(1)$ radius. Here, $\vec{x}_j \in \mathbb{R}^q, q = \mathcal{O}(1)$ and x is the concatenation of L vectors $\vec{x}_1, \ldots, \vec{x}_L$ with dimension $m = Lq = \mathcal{O}(n)$. Individual terms $h_j(\vec{x}_j)$ obey $\|h_j(\vec{x}_j)\|_\infty \leq 1$ and also have bounded directional derivative: $\|\partial h_j/\partial \hat{u}\|_\infty \leq 1$, where $\hat{u}$ is a unit vector in parameter space.

(c) *Ground-state subspace:* We consider the ground state $\rho(x)$ for the Hamiltonian $H(x)$ to be defined as $\rho(x) = \lim_{\beta\to\infty} e^{-\beta H(x)}/\,\mathrm{Tr}\big(e^{-\beta H(x)}\big)$. This is equivalent to a uniform mixture over the eigenspace of $H(x)$ with the minimum eigenvalue.

(d) *Observable:* O can be written as a sum of few-body observables $O = \sum_j O_j$, where each O_j only acts on an $\mathcal{O}(1)$ number of sites. Hence, we can also write $O = \sum_{P \in S^{(\mathrm{geo})}} \alpha_P P$, where $P \in \{I, X, Y, Z\}^{\otimes n}$ and $S^{(\mathrm{geo})}$ is the set of geometrically local Pauli observables (defined more precisely in Def. 6). The results in this section hold for any O of the above form. However, we only focus on O given as a sum of geometrically local observables $\sum_j O_j$, where each O_j only acts on an $\mathcal{O}(1)$ number of sites in a ball of $\mathcal{O}(1)$ radius.

Under these assumptions, we can prove that $\mathrm{Tr}(O\rho(x))$ can be approximated by a sum of weighted indicator functions, where the weights satisfy a ℓ_1-norm bound. A precise statement of this result is found in Section I C.

We first show that $\mathrm{Tr}(O\rho(x))$ can be approximated by a sum of smooth local functions in Section I A. Then, we prove that this sum of smooth local functions can be approximated by simple functions in Section I B. Finally, we put everything together in Section I C. Several technical lemmas for bounding integrals are needed throughout these proofs, which are compiled in Section I D.

A. Approximation by a sum of smooth functions

The key intermediate step is to approximate $\mathrm{Tr}(O\rho(x))$ by a sum of smooth local functions. The proof of this relies on the spectral flow formalism [2] and Lieb-Robinson bounds [3].

First, we review the tools necessary from spectral flow [2, 4, 5]. Let the spectral gap of $H(x)$ be lower bounded by a constant γ over $[-1, 1]^m$. Then, the directional derivative of an associated ground state in the direction defined by the parameter unit vector $\hat{u}$ is given by

$$\frac{\partial \rho}{\partial \hat{u}}(x) = -i[D_{\hat{u}}(x), \rho(x)], \tag{S1}$$

where $D_{\hat{u}}(x)$ is given by

$$D_{\hat{u}}(x) = \int_{-\infty}^{+\infty} W_\gamma(t) e^{itH(x)} \frac{\partial H}{\partial \hat{u}}(x) e^{-itH(x)}\, dt. \tag{S2}$$

Here, $W_\gamma(t)$ is defined by

$$|W_\gamma(t)| \leq \begin{cases} \frac{1}{2} & 0 \leq \gamma|t| \leq \theta, \\ 35e^2(\gamma|t|)^4 e^{-\frac{2}{7}\frac{\gamma|t|}{\log(\gamma|t|)^2}} & \gamma|t| > \theta, \end{cases} \tag{S3}$$

where θ is chosen to be the largest real solution of $35e^2\theta^4 \exp\!\left(-\frac{2}{7}\frac{\theta}{\log^2(\theta)}\right) = 1/2$. Notice that $W_\gamma(t)$ has the property that $\sup_t |W_\gamma(t)| = 1/2$.

Next, we review the Lieb-Robinson bounds [3, 6]. Let the distance $d_{\text{obs}}(X_1, X_2)$ between any two operators X_1, X_2 be defined as the minimum distance between all pairs of sites acted on by X_1 and X_2, respectively, in the d-dimensional space. Formally, this is defined as

$$d_{\text{obs}}(X_1, X_2) \triangleq \min_{\substack{i \in \mathsf{dom}(X_1) \\ i' \in \mathsf{dom}(X_2)}} d_{\text{qubit}}(i, i'), \tag{S4}$$

where $\mathsf{dom}(O)$ contains the qubits that the observable O acts on and $d_{\text{qubit}}(i, i')$ is the distance between two qubits i and i'. Furthermore, notice that for any operator X acting on a single site, a ball of radius r around X contains $\mathcal{O}(r^d)$ local terms in d-dimensional space:

$$\sum_{j : d_{\text{obs}}(X, h_j) \leq r} 1 \leq b_d + c_d r^d, \tag{S5}$$

where h_j is an interaction term of the Hamiltonian $H = \sum_{j=1}^{L} h_j$. Here, this bound implies the existence of a Lieb-Robinson bound [6, 7] such that for any two operators X_1, X_2 and any $t \in \mathbb{R}$,

$$\begin{aligned} &\|[\exp(itH(x)) X_1 \exp(-itH(x)), X_2]\|_\infty \\ &\leq c_{\text{lr}} \|X_1\|_\infty \|X_2\|_\infty |\mathsf{dom}(X_1)| \exp(-a_{\text{lr}}(d_{\text{obs}}(X_1, X_2) - v_{\text{lr}}|t|)), \end{aligned} \tag{S6}$$

where $a_{\text{lr}}, c_{\text{lr}}, v_{\text{lr}} = \Theta(1)$ are constants. Having reviewed these tools, before stating our result formally, we need to define a quantity that we use throughout the proof.

Definition 1. *Let $1/e > \epsilon > 0$. Consider a family of Hamiltonians $\{H(x) : x \in [-1, 1]^m\}$ in a d-dimensional space. Suppose that the spectral gap of $H(x)$ is lower bounded by a constant γ over $[-1, 1]^m$. Define δ_1 as*

$$\delta_1 \triangleq \max\left(C_{\max} \log^2(1/\epsilon), C_4, C_5, \frac{\max(5900, \alpha, 7(d+11), \theta)}{b} \right), \tag{S7}$$

where we denote $b \triangleq \gamma/2v_{\text{lr}}$ for convenience, and v_{lr} is the constant from the Lieb-Robinson bound in Eq. (S6). Here, $C_{\max} = \max(C_1, C_2, C_3)$, where C_1, C_2, C_3 are constants defined in Lemmas 6, 7, 8. Also, we define C_4 as a constant such that for all $\delta' \geq C_4$,

$$\frac{1}{1 - \frac{77 \log^2(b(\delta'+1))}{b(\delta'+1)}} \leq 2. \tag{S8}$$

Similarly, define C_5 as a constant such that for all $\delta' \geq C_5$,

$$\frac{1}{1 - \frac{7(2d+22) \log^2(b(\delta'+1))}{2b(\delta'+1)}} \leq 2. \tag{S9}$$

Moreover, α is defined such that for all $x \geq b(\alpha+1)$, $35 \log^2 x < x - b$. Finally, θ is chosen to be the largest real solution of

$$35 e^2 \theta^4 \exp\left(-\frac{2}{7} \frac{\theta}{\log^2(\theta)} \right) = \frac{1}{2}. \tag{S10}$$

The existence of C_4, C_5 is guaranteed by noting that as δ' goes to infinity, the inequalities become less than or equal to 2. Similarly, the existence of α is guaranteed by considering $x \to \infty$. Using the quantity δ_1 defined above, we also define the parameters "close to" a given Pauli term P.

Definition 2. *Given δ_1 from Definition 1 and an observable $O = \sum_{P \in S^{(\text{geo})}} \alpha_P P$, for each Pauli term $P \in S^{(\text{geo})}$, we define*

$$I_P \triangleq \left\{ c \in \{1, \ldots, m\} : d_{\text{obs}}(h_{j(c)}, P) \leq \delta_1 \right\}, \tag{S11}$$

as in Eq. (II.1) in the main text.

Now, we are ready to present the precise statement that the ground state property $\text{Tr}(O\rho(x))$ can be approximated by a sum of smooth local functions. First, we consider the simpler case where our observable $O = \alpha_P P$ is a single Pauli term, which easily generalizes to the general case via triangle inequality.

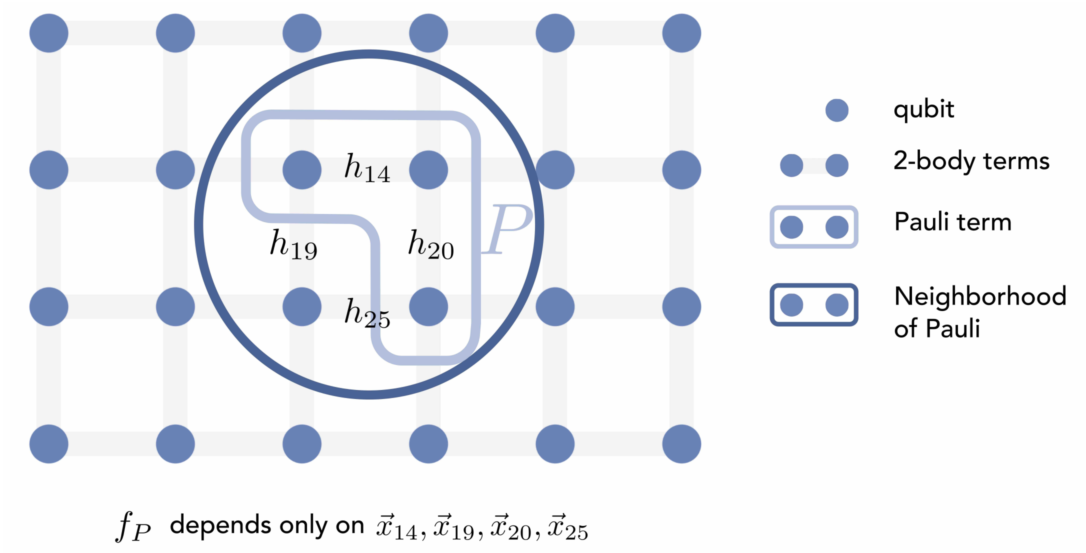

Figure S1: **Intuition behind Lemma 2**. The qubits (blue circles) are arranged in a two-dimensional lattice with local Hamiltonian terms (light gray shading) acting between all pairs of neighboring qubits. A Pauli term P acts on a subset of these qubits indicated by the light blue region. The dark blue circle represents a neighborhood around the region on which P acts. The idea of Lemma 2 is that when changing the parameters x, only $\vec{x}_j$ such that $h_j(\vec{x}_j)$ within the neighborhood around the region that P acts on should significantly change $\mathrm{Tr}(P\rho(x))$. Hence, f_P depends only on those parameters. It is implicit in the figure that h_j depends on $\vec{x}_j$ for all j. Hence, f_P depends only on the variables included in the vectors $\vec{x}_{14}, \vec{x}_{19}, \vec{x}_{20}, \vec{x}_{25}$. In particular, I_P in Def. 2 contains exactly those indices c of the variables x_c that are included in $\vec{x}_{14}, \vec{x}_{19}, \vec{x}_{20}, \vec{x}_{25}$.

Lemma 2 (Approximation using smooth local functions; simple case)**.** *Consider a class of local Hamiltonians $\{H(x) : x \in [-1,1]^m\}$ satisfying assumptions (a)-(c), and an observable $O = \alpha_P P$, where P acts on at most $\mathcal{O}(1)$ qubits. Then, there exists a constant $C > 0$ such that for any $1/e > \epsilon > 0$,*

$$|\alpha_P \mathrm{Tr}(P\rho(x)) - f_P(x)| \leq C|\alpha_P|\epsilon, \tag{S12}$$

where $f_P(x) \triangleq \alpha_P \mathrm{Tr}(P\rho(\chi_P(x)))$ is a smooth function that only depends on parameters $x_c \in [-1,1]$ for coordinates $c \in I_P$, the restriction function $\chi_P : [-1,1]^m \mapsto [-1,1]^m$ is defined as

$$\chi_P(x)_c = \begin{cases} x_c, & c \in I_P, \\ 0, & c \notin I_P, \end{cases} \quad \forall c \in \{1,\ldots,m\}, \tag{S13}$$

and the set I_P of coordinates is given in Definition 2. The function $f_P(x)$ is smooth in the sense that

$$\|\nabla_x f_P(x)\|_2^2 \leq |\alpha_P|^2 C' \tag{S14}$$

for some constant $C' > 0$.

Corollary 2 (Approximation using smooth local functions; general case)**.** *Consider a class of local Hamiltonians $\{H(x) : x \in [-1,1]^m\}$ and an observable $O = \sum_{P \in \{I,X,Y,Z\}^{\otimes n}} \alpha_P P$ satisfying assumptions (a)-(d). There exists a constant $C > 0$ such that for any $1/e > \epsilon > 0$,*

$$|\mathrm{Tr}(O\rho(x)) - f(x)| \leq C\epsilon \left(\sum_P |\alpha_P| \right), \tag{S15}$$

where $f(x) = \sum_{P \in S^{(\mathrm{geo})}} f_P(x)$ for $f_P(x)$ given in Lemma 2.

We illustrate the intuition for Lemma 2 in Figure S1. The proof of Lemma 2 requires several steps. The main idea is that the function $f_P(x)$ is simply $\alpha_P \mathrm{Tr}(P\rho(\chi_P(x)))$ such that $\chi_P(x)_c = x_c$ for $c \in I_P$ and $\chi_P(x)_c = 0$ for coordinates $c \notin I_P$. Thus, we need to show that changing coordinates outside of I_P does not change $\alpha_P \mathrm{Tr}(P\rho(x))$ by much. First, we change one coordinate outside of I_P at a time and show that the directional derivative of $\alpha_P \mathrm{Tr}(P\rho(x))$ in the direction changing this coordinate is bounded. Next, we use this to prove that $|\alpha_P \mathrm{Tr}(P\rho(x)) - \alpha_P \mathrm{Tr}(P\rho(x'))|$ is bounded, where x and x'

differ in this one coordinate. Finally, we show that the difference is bounded for the case where x and x' differ for all coordinates outside of I_P, which concludes the proof of Lemma 2. We separate these results into lemmas. Throughout the proofs of these lemmas, we also need several technical lemmas for showing the existence of certain constants and bounding integrals, proofs of which we relegate to Section I D. In the rest of this section, and in Section I D, we use the notation $b \triangleq \gamma/(2v_{\mathrm{lr}})$ and $\Delta(j,P) \triangleq d_{\mathrm{obs}}(h_{j(c)},P)$ for convenience.

Lemma 3 (Change one coordinate; directional derivative)**.** *Consider a class of local Hamiltonians $\{H(x) : x \in [-1,1]^m\}$ satisfying assumptions (a)-(c), and an observable $O = \alpha_P P$, where P acts on at most $\mathcal{O}(1)$ qubits. Suppose that some $x, x' \in [-1,1]^m$ only differ in one coordinate, say the coordinate c^* such that $c^* \notin I_P$ and only one h_j depends on x_{c^*}. Let $\hat{u}$ be a unit vector in the direction that moves from x to x' along the c^*th coordinate. Then, there exist constants c_1, c_2 such that*

$$\begin{aligned} &|\alpha_P|\,|\hat{u}\cdot\nabla_x \operatorname{Tr}(P\rho(x))| \\ &\leq |\alpha_P| \left(c_1 e^{-\frac{a_{\mathrm{lr}}\Delta(j,P)}{2}} + c_2 \left(\frac{1}{1-\frac{35\log^2(b\Delta(j,P))}{b\Delta(j,P)}} \right) \Delta(j,P)^{10} \exp\left(-\frac{2}{7}\frac{b\Delta(j,P)}{\log^2(b\Delta(j,P))} \right) \right). \end{aligned} \tag{S16}$$

Proof. For the direction $\hat{u}$, we can write the directional derivative of $\rho(x)$ in two ways. First, we have the standard definition:

$$\frac{\partial \rho}{\partial \hat{u}}(x) = \hat{u}\cdot\nabla_x \rho(x). \tag{S17}$$

Then, from spectral flow, we also have Eq. (S1). When evaluated on an observable $O = \alpha_P P$, this establishes the following correspondence:

$$\alpha_P(\hat{u}\cdot\nabla_x \operatorname{Tr}(P\rho(x))) = i\alpha_P \operatorname{Tr}(P[D_{\hat{u}}(x),\rho(x)]) = i\alpha_P \operatorname{Tr}([P, D_{\hat{u}}(x)]\rho(x)). \tag{S18}$$

Expanding $D_{\hat{u}}(x)$ according to Eq. (S2) and applying the triangle inequality to

$$H(x) = \sum_{j=1}^{L} h_j(\vec{x}_j), \tag{S19}$$

we have

$$|\alpha_P||\operatorname{Tr}([P, D_{\hat{u}}(x)]\rho(x))| \leq |\alpha_P| \int_{-\infty}^{+\infty} W_\gamma(t) \sum_{j=1}^{L} \left\| \left[P, e^{itH(x)} \frac{\partial h_j}{\partial \hat{u}} e^{-itH(x)} \right] \right\|_\infty dt. \tag{S20}$$

Here, since x_{c^*} only affects h_j for one j and $\hat{u}$ is in the direction where only the coordinate c^* changes, then

$$\frac{\partial h_{j'}}{\partial \hat{u}} = 0 \tag{S21}$$

for all $j' \neq j$. Thus, we are left with

$$|\alpha_P||\operatorname{Tr}([P, D_{\hat{u}}(x)]\rho(x))| \leq |\alpha_P| \int_{-\infty}^{+\infty} W_\gamma(t) \left\| \left[P, e^{itH(x)} \frac{\partial h_j}{\partial \hat{u}} e^{-itH(x)} \right] \right\|_\infty dt. \tag{S22}$$

We bound this integral using Lieb-Robinson bounds and the inequality on $W_\gamma(t)$ that

$$\sup_t |W_\gamma(t)| = 1/2. \tag{S23}$$

We first need to split the integral into cases. This is because Lieb-Robinson bounds only apply outside of the lightcone, i.e., when $\Delta(j,P) > v_{\mathrm{lr}}|t|$. Then, when $\Delta(j,P) \leq v_{\mathrm{lr}}|t|$, we can instead use the commutator norm bound $\|[A,B]\|_\infty \leq 2\|A\|_\infty \|B\|_\infty$. Thus, we define $t^* = \Delta(j,P)/(2v_{\mathrm{lr}})$ and split up the integration into two parts: $t \in [-t^*, t^*]$ and $t \notin [-t^*, t^*]$ so that we have

$$\begin{aligned} |\alpha_P||\operatorname{Tr}([P, D_{\hat{u}}(x)]\rho(x))| \leq{} & |\alpha_P| \int_{-t^*}^{t^*} W_\gamma(t) \left\| \left[P, e^{itH(x)} \frac{\partial h_j}{\partial \hat{u}} e^{-itH(x)} \right] \right\|_\infty dt \\ &+ |\alpha_P| \int_{t^*}^{+\infty} W_\gamma(t) \left\| \left[P, e^{itH(x)} \frac{\partial h_j}{\partial \hat{u}} e^{-itH(x)} \right] \right\|_\infty dt \\ &+ |\alpha_P| \int_{-\infty}^{-t^*} W_\gamma(t) \left\| \left[P, e^{itH(x)} \frac{\partial h_j}{\partial \hat{u}} e^{-itH(x)} \right] \right\|_\infty dt. \end{aligned} \tag{S24}$$

Notice that the first integral corresponds to the case when we are outside of the lightcone, i.e., $\Delta(j,P) > 2v_{\rm lr}|t|$ while the other two integrals correspond to the case when we are inside of the light cone.

First, we bound the first integral using the Lieb-Robinson bound. Applying Eq. (S6) to the commutator norm, we have

$$\left\|\left[P, e^{itH(x)}\frac{\partial h_j}{\partial \hat{u}}e^{-itH(x)}\right]\right\|_\infty \leq c_{\rm lr}\|P\|_\infty\left\|\frac{\partial h_j}{\partial \hat{u}}\right\|_\infty |\mathsf{dom}(h_j)| e^{-a_{\rm lr}(\Delta(j,P)-v_{\rm lr}|t|)} \tag{S25a}$$

$$\leq c_{\rm lr}c_h e^{-a_{\rm lr}(\Delta(j,P)-v_{\rm lr}|t|)}, \tag{S25b}$$

where in the last inequality, we are using assumption (b) that $\|\partial h_j/\partial \hat{u}\|_\infty \leq 1$ and $|\mathsf{dom}(h_j)| \leq c_h$ for a constant c_h. Plugging this into the integral, we have

$$|\alpha_P|\int_{-t^*}^{t^*} W_\gamma(t)\left\|\left[P, e^{itH(x)}\frac{\partial h_j}{\partial \hat{u}}e^{-itH(x)}\right]\right\|_\infty dt \leq |\alpha_P| c_{\rm lr}c_h e^{-a_{\rm lr}\Delta(j,P)}\int_{-t^*}^{t^*}|W_\gamma(t)|e^{a_{\rm lr}v_{\rm lr}|t|}\,dt \tag{S26a}$$

$$\leq \frac{1}{2}|\alpha_P| c_{\rm lr}c_h e^{-a_{\rm lr}\Delta(j,P)}\int_{-t^*}^{t^*} e^{a_{\rm lr}v_{\rm lr}|t|}\,dt \tag{S26b}$$

$$= |\alpha_P| c_{\rm lr}c_h e^{-a_{\rm lr}\Delta(j,P)}\int_{0}^{t^*} e^{a_{\rm lr}v_{\rm lr}t}\,dt \tag{S26c}$$

$$= |\alpha_P|\frac{c_{\rm lr}c_h e^{-a_{\rm lr}\Delta(j,P)}}{a_{\rm lr}v_{\rm lr}}\left(e^{a_{\rm lr}v_{\rm lr}t^*}-1\right) \tag{S26d}$$

$$= |\alpha_P|\frac{c_{\rm lr}c_h}{a_{\rm lr}v_{\rm lr}}e^{-a_{\rm lr}\Delta(j,P)}\left(e^{a_{\rm lr}\Delta(j,P)/2}-1\right) \tag{S26e}$$

$$= |\alpha_P|\frac{c_{\rm lr}c_h}{a_{\rm lr}v_{\rm lr}}\left(e^{-a_{\rm lr}\Delta(j,P)/2}-e^{-a_{\rm lr}\Delta(j,P)}\right) \tag{S26f}$$

$$\leq |\alpha_P|\frac{c_{\rm lr}c_h}{a_{\rm lr}v_{\rm lr}}e^{-a_{\rm lr}\Delta(j,P)/2}, \tag{S26g}$$

where in the second line, we used the fact that $\sup_t |W_\gamma(t)| = 1/2$, and in the fifth line, we substituted back in $t^* = \Delta(j,P)/(2v_{\rm lr})$.

We can also bound the other integrals using the commutator norm bound

$$\|[A,B]\|_\infty \leq 2\|A\|_\infty\|B\|_\infty \tag{S27}$$

to obtain:

$$|\alpha_P|\int_{t^*}^{+\infty} W_\gamma(t)\left\|\left[P, e^{itH(x)}\frac{\partial h_j}{\partial \hat{u}}e^{-itH(x)}\right]\right\|_\infty dt \leq 2|\alpha_P|\int_{t^*}^{+\infty}|W_\gamma(t)|\|P\|_\infty\left\|\frac{\partial h_j}{\partial \hat{u}}\right\|_\infty dt \tag{S28a}$$

$$\leq 2|\alpha_P|\int_{t^*}^{\infty}|W_\gamma(t)|\,dt, \tag{S28b}$$

where in the second line, we used assumption (b) that $\|\partial h_j/\partial \hat{u}\|_\infty \leq 1$. To bound the resulting integral, we use the definition of $W_\gamma(t)$ in Eq. (S3). Note that by our definition of t^*, $\gamma t^* > \theta$, so we only need to consider this case in the upper bound on $W_\gamma(t)$. This is because we chose

$$\delta_1 = \max\left(C_{\max}\log^2(1/\epsilon), C_4, C_5, \frac{\max(5900,\alpha,7(d+11),\theta)}{b}\right), \tag{S29}$$

and here we consider $\Delta(j,P) > \delta_1$. Thus, we have

$$\gamma t^* = \frac{\gamma\Delta(j,P)}{2v_{\rm lr}} > \frac{\gamma\delta_1}{2v_{\rm lr}} \geq \max(5900,\alpha,7(d+11),\theta) \geq \theta. \tag{S30}$$

Hence, we can bound the integral:

$$\int_{t^*}^{+\infty}|W_\gamma(t)|\,dt \leq 35e^2\int_{t^*}^{+\infty}(\gamma t)^4 e^{-\frac{2}{7}\frac{\gamma t}{\log^2(\gamma t)}}\,dt = 35e^2\gamma^{-1}\int_{x=\gamma t^*}^{+\infty}x^4 e^{-\frac{2}{7}\frac{x}{\log^2(x)}}\,dx. \tag{S31}$$

In the inequality, we used the definition of $W_\gamma(t)$ and in the equality, we used the substitution $x = \gamma t$. We can bound this integral using Lemma 9. Set $a = 2/7$ and $k = 4$. We have chosen t^* and δ_1 such that all of the assumptions of Lemma 9 are satisfied. In particular, from Eq. (S30), we see that

$t = \gamma t^* > \max(5900, \alpha, 7(d+11), \theta) \geq 5900$. Furthermore, we have $at/\log^2(t) > 2k+2$, because if $t \geq 5900$, then it is clear that $at/\log^2(t) > 10$. Now, applying Lemma 9, we have

$$\int_{t^*}^{+\infty} |W_\gamma(t)|\, dt \leq \frac{245}{2} e^2 \gamma^{-1} \left(\frac{1}{1 - \frac{35\log^2(\gamma t^*)}{\gamma t^*}} \right) (\gamma t^*)^{10} e^{-\frac{2}{7}\frac{\gamma t^*}{\log^2(\gamma t^*)}}. \tag{S32}$$

The last integral can be bounded in exactly the same way. Plugging these bounds into Eq. (S24), we have

$$|\alpha_P| |\operatorname{Tr}([P, D_{\hat{u}}(x)]\rho(x))| \tag{S33a}$$

$$\leq |\alpha_P| \left(\frac{c_{\mathrm{lr}} c_h}{a_{\mathrm{lr}} v_{\mathrm{lr}}} e^{-a_{\mathrm{lr}}\Delta(j,P)/2} + 4\left(\frac{245}{2} e^2 \gamma^{-1} \left(\frac{1}{1 - \frac{35\log^2(\gamma t^*)}{\gamma t^*}} \right) (\gamma t^*)^{10} e^{-\frac{2}{7}\frac{\gamma t^*}{\log^2(\gamma t^*)}} \right) \right) \tag{S33b}$$

$$= |\alpha_P| \left(c_1 e^{-a_{\mathrm{lr}}\Delta(j,P)/2} + c_2 \left(\frac{1}{1 - \frac{35\log^2(b\Delta(j,P))}{b\Delta(j,P)}} \right) \Delta(j,P)^{10} \exp\left(-\frac{2}{7} \frac{b\Delta(j,P)}{\log^2(b\Delta(j,P))} \right) \right), \tag{S33c}$$

where in the second line, we defined constants

$$c_1 = \frac{c_{\mathrm{lr}} c_h}{a_{\mathrm{lr}} v_{\mathrm{lr}}}, \quad c_2 = \frac{245 e^2 b^9}{v_{\mathrm{lr}}}. \tag{S34}$$

Thus, we have proven that if we only change one coordinate outside of I_P, then the directional derivative changing this coordinate is small. This is exactly the claim of the lemma. □

An immediate consequence of this is that we can integrate the directional derivative to obtain a bound on the distance between $\operatorname{Tr}(P\rho(x))$ and $\operatorname{Tr}(P\rho(x'))$.

Lemma 4 (Change one coordinate; distance)**.** *Consider a class of local Hamiltonians $\{H(x) : x \in [-1,1]^m\}$ satisfying assumptions (a)-(c), and an observable $O = \alpha_P P$, where P acts on at most $\mathcal{O}(1)$ qubits. Suppose that some $x, x' \in [-1,1]^m$ only differ in one coordinate, say the coordinate c^* such that $c^* \notin I_P$ and only one h_j depends on x_{c^*}. Let $\hat{u}$ be a unit vector in the direction that moves from x to x' along the c^*th coordinate. Then, there exist constants c_1', c_2' such that*

$$\begin{aligned} &|\alpha_P| |\operatorname{Tr}(P\rho(x)) - \operatorname{Tr}(P\rho(x'))| \\ &\leq |\alpha_P| \left(c_1' e^{-\frac{a_{\mathrm{lr}}\Delta(j,P)}{2}} + c_2' \left(\frac{1}{1 - \frac{35\log^2(b\Delta(j,P))}{b\Delta(j,P)}} \right) \Delta(j,P)^{10} \exp\left(-\frac{2}{7} \frac{b\Delta(j,P)}{\log^2(b\Delta(j,P))} \right) \right). \end{aligned} \tag{S35}$$

Proof. By Lemma 3, we have a bound on the directional derivative of $\alpha_P \operatorname{Tr}(P\rho(x))$ in the direction of $\hat{u}$. In this lemma, we want a bound on the distance

$$|\alpha_P| |\operatorname{Tr}(P\rho(x)) - \operatorname{Tr}(P\rho(x'))| = |\alpha_P| |\operatorname{Tr}(P\rho(x_1, \ldots, x_{c^*}, \ldots, x_m)) - \operatorname{Tr}(P\rho(x_1, \ldots, x'_{c^*}, \ldots, x_m))|. \tag{S36}$$

To this end, we can obtain the distance by integrating the directional derivative:

$$|\alpha_P| |\operatorname{Tr}(P\rho(x)) - \operatorname{Tr}(P\rho(x'))| \tag{S37a}$$

$$= |\alpha_P| \left| \int_{x_{c^*}}^{x'_{c^*}} \frac{\partial \operatorname{Tr}(P\rho(x_1, \ldots, t, \ldots, x_m))}{\partial \hat{u}}\, dt \right| \tag{S37b}$$

$$\leq |\alpha_P| \int_{x_{c^*}}^{x'_{c^*}} \left| \frac{\partial \operatorname{Tr}(P\rho(x_1, \ldots, t, \ldots, x_m))}{\partial \hat{u}} \right| dt \tag{S37c}$$

$$= |\alpha_P| \int_{x_{c^*}}^{x'_{c^*}} |\operatorname{Tr}([P, D_{\hat{u}}(x_1, \ldots, t, \ldots, x_m)]\rho(x_1, \ldots, t, \ldots, x_m))|\, dt, \tag{S37d}$$

where in the last line, we used the correspondence from Eq. (S18). Now, the integrand is exactly what we bounded in Lemma 3, so we have

$$|\alpha_P| |\operatorname{Tr}(P\rho(x)) - \operatorname{Tr}(P\rho(x'))| \tag{S38a}$$

$$\leq |\alpha_P| \int_{x_{c^*}}^{x'_{c^*}} \left(c_1 e^{-a_{\mathrm{lr}}\Delta(j,P)/2} + c_2 \left(\frac{1}{1 - \frac{35\log^2(b\Delta(j,P))}{b\Delta(j,P)}} \right) \Delta(j,P)^{10} \exp\left(-\frac{2}{7} \frac{b\Delta(j,P)}{\log^2(b\Delta(j,P))} \right) \right) dt \tag{S38b}$$

$$\leq 2|\alpha_P| \left(c_1 e^{-a_{\mathrm{lr}}\Delta(j,P)/2} + c_2 \left(\frac{1}{1 - \frac{35\log^2(b\Delta(j,P))}{b\Delta(j,P)}} \right) \Delta(j,P)^{10} \exp\left(-\frac{2}{7} \frac{b\Delta(j,P)}{\log^2(b\delta_1)} \right) \right), \tag{S38c}$$

where in the last line, we can bound this integral because $x_{c^*}, x'_{c^*} \in [-1,1]$, so their difference is at most 2. Taking $c'_1 = 2c_1$ and $c'_2 = 2c_2$, we arrive at the claim. □

With these two results, we can prove Lemma 2.

Proof of Lemma 2. It remains to show that if we change multiple coordinates outside of I_P, the difference $|\operatorname{Tr}(P\rho(x)) - \operatorname{Tr}(P\rho(x'))|$ is still bounded. Then, taking $f_P(x)$ to be $\alpha_P \operatorname{Tr}(P\rho(\chi_P(x)))$ with $\chi_P(x) \in [-1,1]^m$ equal to x_c for coordinates $c \in I_P$ and 0 for coordinates outside of I_P gives the desired result. Thus, we want to bound

$$|\alpha_P||\operatorname{Tr}(P\rho(x)) - \operatorname{Tr}(P\rho(x'))| = |\alpha_P||\operatorname{Tr}(P\rho(x_1,\ldots,x_m)) - \operatorname{Tr}(P\rho(x'_1,\ldots,x'_m))|, \tag{S39}$$

where $x'_{c'} = x_{c'}$ if and only if $c' \in I_P$. We can bound this using the triangle inequality

$$\begin{aligned}
|\alpha_P||\operatorname{Tr}(P\rho(x)) - \operatorname{Tr}(P\rho(x'))| \leq\ & |\alpha_P||\operatorname{Tr}(P\rho(x_1,x_2,\ldots,x_m)) - \operatorname{Tr}(P\rho(x'_1,x_2,\ldots,x_m))| \\
&+ |\alpha_P||\operatorname{Tr}(P\rho(x'_1,x_2,\ldots,x_m)) - \operatorname{Tr}(P\rho(x'_1,x'_2,\ldots,x_m))| \\
&+ \cdots \\
&+ |\alpha_P||\operatorname{Tr}\big(P\rho(x'_1,\ldots,x'_{m-1},x_m)\big) - \operatorname{Tr}(P\rho(x'_1,\ldots,x'_m))|
\end{aligned} \tag{S40}$$

Here, recall that we are only changing coordinates outside of I_P, i.e., x_c such that h_j depends on x_c and $\Delta(j,P) > \delta_1$ for δ_1 in Definition 1. Moreover, by assumption (b), each local term h_j depends on at most a constant number q of parameters. Then, we can upper bound this sum by

$$|\alpha_P||\operatorname{Tr}(P\rho(x)) - \operatorname{Tr}(P\rho(x'))| \leq q|\alpha_P| \sum_{j:\Delta(j,P)>\delta_1} |\operatorname{Tr}(P\rho(y_{k^*})) - \operatorname{Tr}(P\rho(y'_{k^*}))|, \tag{S41a}$$

where k^* is defined as

$$k^* \triangleq \operatorname*{arg\,max}_{1\leq k\leq m} |\operatorname{Tr}(P\rho(y_k)) - \operatorname{Tr}(P\rho(y'_k))|, \tag{S42}$$

and $y_k, y'_k \in [-1,1]^m$ denote parameter vectors that only differ in the kth coordinate. Each of these terms in the summand can be bounded using Lemma 4:

$$|\alpha_P||\operatorname{Tr}(P\rho(x)) - \operatorname{Tr}(P\rho(x'))| \tag{S43a}$$

$$\leq q|\alpha_P| \sum_{j:\Delta(j,P)>\delta_1} \left(c'_1 e^{-\frac{a_{\mathrm{lr}}\Delta(j,P)}{2}} + c'_2 \left(\frac{1}{1 - \frac{35\log^2(b\Delta(j,P))}{b\Delta(j,P)}} \right) \Delta(j,P)^{10} \exp\left(-\frac{2}{7} \frac{b\Delta(j,P)}{\log^2(b\Delta(j,P))} \right) \right) \tag{S43b}$$

$$\begin{aligned}
\leq q|\alpha_P| \sum_{r=0}^{\infty} \sum_{j:\Delta(j,P)\in[\delta_1+r,\delta_1+r+1]} \Bigg(& c'_1 e^{-\frac{a_{\mathrm{lr}}\Delta(j,P)}{2}} \\
&+ c'_2 \left(\frac{1}{1 - \frac{35\log^2(b\Delta(j,P))}{b\Delta(j,P)}} \right) \Delta(j,P)^{10} \exp\left(-\frac{2}{7} \frac{b\Delta(j,P)}{\log^2(b\Delta(j,P))} \right) \Bigg).
\end{aligned} \tag{S43c}$$

Now, we want to upper bound this inner sum over j such that $\Delta(j,P) \in [\delta_1+r, \delta_1+r+1]$. Using Eq. (S5), we see that there are at most $|\mathsf{dom}(P)|(b_d + c_d(\delta_1+r+1)^d)$ interaction terms h_j such that $\Delta(j,P) \in [\delta_1+r, \delta_1+r+1]$. Moreover, because P acts on only a constant number of sites (by assumption), then

$$|\mathsf{dom}(P)|(b_d + c_d(\delta_1+r+1)^d) \leq c_P(b_d + c_d(\delta_1+r+1)^d), \tag{S44}$$

for some constant c_P. Using this as well as upper bounding the sum over r by an integral, we have

$$|\alpha_P||\operatorname{Tr}(P\rho(x)) - \operatorname{Tr}(P\rho(x'))| \tag{S45a}$$

$$\leq qc_P|\alpha_P| \int_{r=0}^{+\infty} (b_d + c_d(\delta_1 + r + 1)^d) \tag{S45b}$$

$$\cdot \left(c_1' e^{-\frac{a_{\mathrm{lr}}(\delta_1+r)}{2}} + c_2' \left(\frac{1}{1 - \frac{35\log^2(b(\delta_1+r+1))}{b(\delta_1+r)}} \right) (\delta_1 + r + 1)^{10} \exp\left(-\frac{2}{7} \frac{b(\delta_1 + r)}{\log^2(b(\delta_1 + r + 1))} \right) \right) dr. \tag{S45c}$$

It remains to integrate this to obtain our desired bound. Distributing, we can split this integral into four terms. We bound each of these individually.

First, we have

$$\int_{r=0}^{+\infty} b_d c_1' e^{-a_{\mathrm{lr}}(\delta_1+r)/2}\, dr = c_1' b_d e^{-a_{\mathrm{lr}}\delta_1/2} \int_{r=0}^{+\infty} e^{-a_{\mathrm{lr}} r/2}\, dr = \frac{2c_1' b_d}{a_{\mathrm{lr}}} e^{-a_{\mathrm{lr}}\delta_1/2}. \tag{S46}$$

We also have

$$\int_{r=0}^{+\infty} c_d c_1' (\delta_1 + r + 1)^d e^{-a_{\mathrm{lr}}(\delta_1+r)/2}\, dr = c_1' c_d e^{-a_{\mathrm{lr}}\delta_1/2} \int_{r=0}^{+\infty} (\delta_1 + r + 1)^d e^{-a_{\mathrm{lr}} r/2}\, dr \tag{S47a}$$

$$= c_1' c_d e^{-a_{\mathrm{lr}}\delta_1/2} \sum_{k=0}^{d} \frac{d!\, 2^{d-k+1}}{k!\, a_{\mathrm{lr}}^{d-k+1}} (\delta_1 + 1)^k, \tag{S47b}$$

where in the last equality we used integration by parts. For the other two integrals, we use Lemma 11 to obtain

$$\int_{r=0}^{+\infty} b_d c_2' \left(\frac{1}{1 - \frac{35\log^2(b(\delta_1+r+1))}{b(\delta_1+r)}} \right) (\delta_1 + r + 1)^{10} \exp\left(-\frac{2}{7} \frac{b(\delta_1 + r)}{\log^2(b(\delta_1 + r + 1))} \right) dr \leq c\epsilon, \tag{S48}$$

for some constant c. Similarly, for the last integral, by Lemma 12, we have

$$\int_{r=0}^{+\infty} c_d c_2' \left(\frac{1}{1 - \frac{35\log^2(b(\delta_1+r+1))}{b(\delta_1+r)}} \right) (\delta_1 + r + 1)^{d+10} \exp\left(-\frac{2}{7} \frac{b(\delta_1 + r)}{\log^2(b(\delta_1 + r + 1))} \right) dr \leq c'\epsilon, \tag{S49}$$

for some constant c'. Putting everything together, we have

$$|\alpha_P||\operatorname{Tr}(P\rho(x)) - \operatorname{Tr}(P\rho(x'))| \tag{S50a}$$

$$\leq qc_P|\alpha_P| \left(\frac{2c_1' b_d}{a_{\mathrm{lr}}} e^{-a_{\mathrm{lr}}\delta_1/2} + c_1' c_d e^{-a_{\mathrm{lr}}\delta_1/2} \sum_{k=0}^{d} \frac{d! 2^{d-k+1}}{k! a_{\mathrm{lr}}^{d-k+1}} (\delta_1 + 1)^k + c\epsilon + c'\epsilon \right). \tag{S50b}$$

Combining constants and simplifying, we have

$$|\alpha_P||\operatorname{Tr}(P\rho(x)) - \operatorname{Tr}(P\rho(x'))| \leq |\alpha_P| \left(e^{-a_{\mathrm{lr}}\delta_1/2} \sum_{k=0}^{d} c_k'' \delta_1^k + c''\epsilon \right). \tag{S51}$$

To obtain the final bound, we can use our choice of δ_1 to write this bound in terms of ϵ:

$$e^{-a_{\mathrm{lr}}\delta_1/2} \sum_{k=0}^{d} c_k'' \delta_1^k = \sum_{k=0}^{d} c_k'' e^{-a_{\mathrm{lr}}\delta_1/2 + k\log\delta_1} \leq \left(\sum_{k=0}^{d} c_k'' \right) \epsilon, \tag{S52}$$

where the last inequality follows from our choice of δ_1 in Definition 1 and C_1 in Lemma 6. Thus, we have

$$|\alpha_P||\operatorname{Tr}(P\rho(x)) - \operatorname{Tr}(P\rho(x'))| \leq C|\alpha_P|\epsilon, \tag{S53}$$

where we take

$$C = \sum_{k=0}^{d} c_k'' + c''. \tag{S54}$$

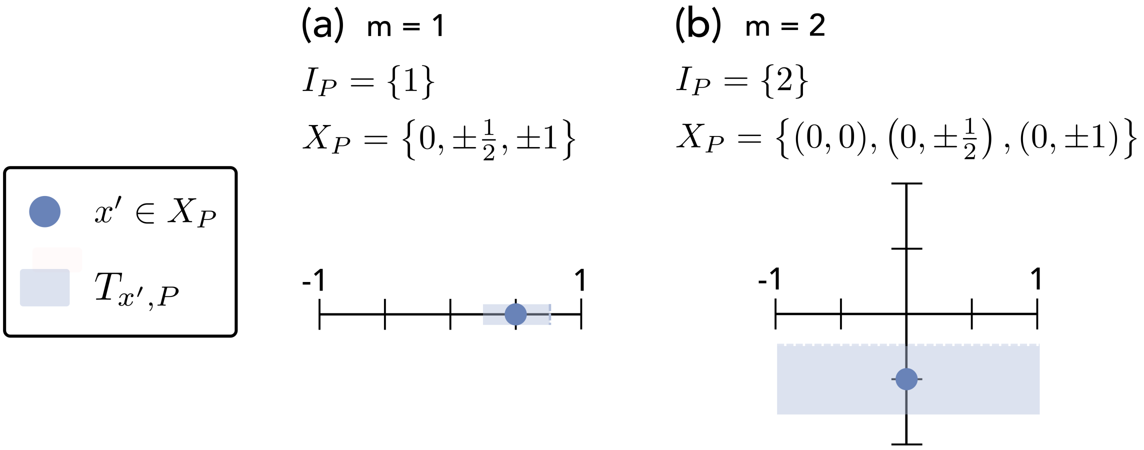

Figure S2: **Example of Definition 3**. Illustration of the set $T_{x',P}$ (light blue shading) for specific $x' \in X_P$ (blue circle), fixing $\delta_2 = 1/2$ for simplicity. **a** Example for $m = 1$. I_P is fixed to $\{1\}$ so that $X_P = \{0, \pm 1/2, \pm 1\}$ according to Def. 3. $T_{x',P}$ is depicted for the chosen $x' = 1/2$. **b** Example for $m = 2$. I_P is fixed to $\{2\}$, and $T_{x',P}$ is depicted for the chosen $x' = (0, -1/2)$.

To complete the proof, recall that $f_P(x) = \alpha_P \operatorname{Tr}(P\rho(\chi_P(x)))$, where χ_P is defined in Eq. (S13). The function f_P only depends on parameters in I_P by definition. By the previous analysis, since $\chi_P(x)$ and x only differs in the coordinates outside of the set I_P, the function $f_P(x)$ should be close to $\alpha_P \operatorname{Tr}(P\rho(\chi_P(x)))$ in absolute value as required. Moreover, $\operatorname{Tr}(P\rho(x))$ is smooth by Lemma 4 in [1] in that

$$\|\nabla_x \operatorname{Tr}(P\rho(x))\|_2^2 \leq C'\|P\|_\infty^2 = C' \tag{S55}$$

for some constant $C' > 0$. Then, because f_P is defined as $\alpha_P \operatorname{Tr}(P\rho(\chi_P(x)))$, we have

$$\|\nabla_x f_P(x)\|_2^2 \leq |\alpha_P|^2 C', \tag{S56}$$

so f_P is smooth as claimed. □

B. Simplification using discretization

Now, we want to show that the sum of smooth local functions $f(x) = \sum_{P \in S^{(\text{geo})}} f_P(x)$ from Corollary 2 can be approximated by simple functions, i.e., linear combinations of indicator functions. In order to do so, we discretize our parameter space and map each $x \in [-1,1]^m$ to some x' with discrete values. Our simple function is then f evaluated on this discretized x'. To state this more precisely, we first require some definitions. An illustrative example of how each set is defined is given in Figure S2.

Definition 3 (Discretization)**.** *Let $\epsilon > 0$. Let*

$$\delta_2 \triangleq \frac{1}{\left\lceil \frac{\sqrt{C'|I_P|}}{\epsilon} \right\rceil}, \tag{S57}$$

where I_P is defined in Definition 2 and C' is as in Lemma 2. Define the discretized parameter space as

$$X_P \triangleq \left\{ \begin{array}{l} x \in [-1,1]^m : \text{if } c \notin I_P, x_c = 0 \\ \qquad\qquad\qquad \text{if } c \in I_P, x_c \in \{0, \pm\delta_2, \pm 2\delta_2, \ldots, \pm 1\} \end{array} \right\}. \tag{S58}$$

Moreover, for each $x \in X_P$, define the thickened affine subspace close to the vector x for coordinates in I_P as

$$T_{x,P} \triangleq \left\{ x' \in [-1,1]^m : -\frac{\delta_2}{2} < x_c - x'_c \leq \frac{\delta_2}{2}, \forall c \in I_P \right\}. \tag{S59}$$

With these definitions, the simple function that approximates f is defined by

$$g(x) \triangleq \sum_{P\in S^{(\mathrm{geo})}} \left[\sum_{x'\in X_P} f_P(x')\mathbb{1}[x\in T_{x',P}]\right] \triangleq \sum_{P\in S^{(\mathrm{geo})}} g_P(x). \tag{S60}$$

In what follows, we prove that g indeed approximates f well. As in Section I A, we first consider the simpler case where our observable $O=\alpha_P P$ is a single Pauli term, which easily generalizes to the general case via triangle inequality.

Lemma 5 (Approximation using simple functions; simple case). *Let $\epsilon>0$. Given this ϵ in Definition 3,*

$$|g_P(x)-f_P(x)| < \epsilon|\alpha_P| \tag{S61}$$

for any x, where f_P is as in Lemma 2 and g_P is defined in Eq. (S60).

Corollary 3 (Approximation using simple functions; general case). *Let $\epsilon>0$. Given this ϵ in Definition 3, then*

$$|g(x)-f(x)| < \epsilon\left(\sum_{P\in S^{(\mathrm{geo})}}|\alpha_P|\right) \tag{S62}$$

for any x, where f is as in Corollary 2 and g is defined in Eq. (S60).

Proof of Lemma 5. Consider some input $x\in[-1,1]^m$. First, we want to argue that $x\in T_{x',P}$ for exactly one $x'\in X_P$. Consider some variable $x_c\in[-1,1]$ of x for $c\in I_P$. It suffices to show that there exists $x'_c\in\{0,\pm\delta_2,\pm2\delta_2,\dots,\pm1\}$ such that $-\delta_2/2 < x'_c - x_c \le \delta_2/2$. This is clear because δ_2 is defined as a fraction of the form $1/n$ for an integer n. Moreover, there is at most one x'_c such that this is true because each possible discrete value of x'_c is separated by intervals of size δ_2 while x_c is within $\delta_2/2$ of x'_c, so there cannot be overlap for different values of x'_c. Also, since x_c is in a half-open interval of x'_c, this prevents points on the boundary (i.e., exactly $\delta_2/2$ away from x'_c) from being associated with two x'_c. Finally, this half-open interval does not prevent the boundary case of $x_c=-1$ from being associated with an x'_c because -1 is always a possible discrete value for x'_c. This occurs again because of our choice of δ_2 as a fraction of the form $1/n$ for an integer n. Thus, $x\in T_{x',P}$ for exactly one $x'\in X_P$.

With this, our goal is to show that

$$|g_P(x)-f_P(x)| = |f_P(x')-f_P(x)| < \epsilon|\alpha_P|. \tag{S63}$$

There are two parts to proving this. By definition of $T_{x',P}$ in Definition 3, this means that x' and x are close for coordinates in I_P. However, for coordinates not in I_P, x' and x can be far away. Nevertheless, from our results in Lemma 2, we know that f_P does not change much when its input only differs for coordinates not in I_P. Thus, we can use this to obtain our bound.

To make this more clear, we introduce the notation

$$f_P(x) = f_P(x_{\mathrm{in}}; x_{\mathrm{out}}), \tag{S64}$$

where x_{in} denotes the variables $x_c\in[-1,1]$ such that $c\in I_P$ and x_{out} denotes the variables $x_c\in[-1,1]$ such that $c\notin I_P$. With this, we can use the triangle inequality to treat the two cases separately:

$$\begin{aligned}
|f_P(x')-f_P(x)| &= |f_P(x'_{\mathrm{in}};x'_{\mathrm{out}}) - f_P(x_{\mathrm{in}};x_{\mathrm{out}})| && \text{(S65a)}\\
&\le |f_P(x'_{\mathrm{in}};x'_{\mathrm{out}}) - f_P(x'_{\mathrm{in}};x_{\mathrm{out}})| \\
&\quad + |f_P(x'_{\mathrm{in}};x_{\mathrm{out}}) - f_P(x_{\mathrm{in}};x_{\mathrm{out}})|. && \text{(S65b)}
\end{aligned}$$

Here, in the first term, only the coordinates not in I_P change while in the second term, only coordinates in I_P change. To bound the first term, we can use Lemma 2 with ϵ set to $\epsilon/(2C)$, where C is the constant defined in Lemma 2, to obtain

$$|f_P(x'_{\mathrm{in}};x'_{\mathrm{out}}) - f_P(x'_{\mathrm{in}};x_{\mathrm{out}})| \le |\alpha_P|\frac{\epsilon}{2}. \tag{S66}$$

For the second term in Eq. (S65), we bound this using the fact that x' and x are separated by at most δ_2 for coordinates in I_P and the smoothness condition on f_P from Lemma 2. The key step here is that we can write this difference as the integral of the directional derivative of f_P along the direction from x_{in} to x'_{in} given by a line. In particular, we can parameterize this line by $x_{\mathrm{in}}(t) = x_{\mathrm{in}} + (x'_{\mathrm{in}} - x_{\mathrm{in}})t$.

Notice that at $t = 0$, this is equal to x_{in} while at $t = 1$, this is equal to x'_{in}. Thus, suppressing the x_{out} parameters in our notation, we have

$$\begin{aligned}
|f_P(x'_{\text{in}}) - f_P(x_{\text{in}})| &= \left| \int_0^1 \frac{\partial f_P(x_{\text{in}}(t))}{\partial t}\, dt \right| && \text{(S67a)}\\
&\leq \int_0^1 \left| \frac{\partial f_P(x_{\text{in}}(t))}{\partial t} \right| dt && \text{(S67b)}\\
&= \int_0^1 \left| \frac{\partial f_P(x_{\text{in}}(t))}{\partial x_{\text{in}}(t)} \cdot \frac{\partial x_{\text{in}}(t)}{\partial t} \right| dt && \text{(S67c)}\\
&= \int_0^1 |\nabla_{x_{\text{in}}} f_P(x_{\text{in}}) \cdot (x'_{\text{in}} - x_{\text{in}})|\, dt && \text{(S67d)}\\
&\leq \int_0^1 \|\nabla_{x_{\text{in}}} f_P(x_{\text{in}})\|_2 \|x'_{\text{in}} - x_{\text{in}}\|_2\, dt && \text{(S67e)}\\
&\leq \sqrt{C'}|\alpha_P| \|x'_{\text{in}} - x_{\text{in}}\|_2 && \text{(S67f)}\\
&\leq \sqrt{C'}|\alpha_P| \sqrt{|I_P|} \|x'_{\text{in}} - x_{\text{in}}\|_\infty && \text{(S67g)}\\
&\leq \sqrt{C'}|\alpha_P| \sqrt{|I_P|} \frac{\delta_2}{2} && \text{(S67h)}\\
&\leq \frac{\epsilon}{2}|\alpha_P|. && \text{(S67i)}
\end{aligned}$$

Here, in the third line, we use the chain rule. In the fifth line, we use the Cauchy-Schwarz inequality. In the sixth line, we use the smoothness condition from Lemma 2 to bound the ℓ_2-norm of the gradient. In the seventh line, we use the fact that $\|y\|_2 \leq \sqrt{n}\|y\|_\infty$ where n is the number of elements in y. In the eighth line, we use the definition of $T_{x,P}$. Finally, in the last line, we use our choice of δ_2 as

$$\delta_2 = \frac{1}{\left\lceil \frac{\sqrt{C'|I_P|}}{\epsilon} \right\rceil} \leq \frac{\epsilon}{\sqrt{C'|I_P|}}. \tag{S68}$$

Combining this bound with Eq. (S66) and plugging into Eq. (S65), we have

$$|f_P(x') - f_P(x)| < \frac{\epsilon}{2}|\alpha_P| + \frac{\epsilon}{2}|\alpha_P| = \epsilon|\alpha_P|, \tag{S69}$$

as required. □

C. Simple form for ground state property

We can combine the results of the previous two sections to obtain the final result giving a simpler form for the ground state property $\text{Tr}(O\rho(x))$. The proof of this statement is simple given the previous results.

Theorem 3 (Simple form for $\text{Tr}(O\rho(x))$). *Let $1/e > \epsilon > 0$. Given ϵ, we define δ_1 according to Definition 1 with ϵ set to $\epsilon/(2C)$ for the constant C defined in Corollary 2, and define δ_2 according to Definition 3 with ϵ set to $\epsilon/2$. The ground state property $\text{Tr}(O\rho(x))$ can be approximated by a simple function, i.e.,*

$$|\text{Tr}(O\rho(x)) - g(x)| < \epsilon \left(\sum_{P \in S^{(\text{geo})}} |\alpha_P| \right), \tag{S70}$$

where g is defined in Eq. (S60).

Proof. By the triangle inequality, we have

$$|\text{Tr}(O\rho(x)) - g(x)| \leq |\text{Tr}(O\rho(x)) - f(x)| + |f(x) - g(x)|. \tag{S71}$$

Here, the first term can be bounded by Corollary 2 to obtain

$$|\text{Tr}(O\rho(x)) - f(x)| \leq \frac{\epsilon}{2} \left(\sum_{P \in S^{(\text{geo})}} |\alpha_P| \right). \tag{S72}$$

Meanwhile, the second term in Eq. (S71) can be bounded by Corollary 3 to obtain

$$|f(x) - g(x)| < \frac{\epsilon}{2}\left(\sum_{P\in S^{(\mathrm{geo})}} |\alpha_P|\right). \tag{S73}$$

Combining Eq. (S72) and Eq. (S73) in Eq. (S71), we have

$$|\operatorname{Tr}(O\rho(x)) - g(x)| < \epsilon\left(\sum_{P\in S^{(\mathrm{geo})}} |\alpha_P|\right) \tag{S74}$$

This concludes the proof. □

D. Technical lemmas for finding constants and bounding integrals

In this section, we state and prove several technical lemmas for showing the existence of certain constants and bounding integrals of specific forms needed throughout Section I. Throughout this section, we use the notation $b \triangleq \gamma/(2v_{\mathrm{lr}})$. First, we show the existence of the constants utilized in Definition 1.

Lemma 6. *Given $a_{\mathrm{lr}}, b > 0$ and $d \geq 1$, there exists a constant C_1 large enough such that for all $1/e > \epsilon' > 0$ and for all $\delta_1' > C_1 \log^2(1/\epsilon')$,*

$$\frac{a_{\mathrm{lr}}}{2}\delta_1' - d\log(\delta_1') \geq \log\left(\frac{1}{\epsilon'}\right). \tag{S75}$$

Explicitly, such a constant C_1 can be given by

$$C_1 = \frac{(2d + \sqrt{4d^2 + 2a_{\mathrm{lr}}})^2}{a_{\mathrm{lr}}^2}. \tag{S76}$$

Proof. For simplicity, throughout this proof, let $x = \log(1/\epsilon')$. Because we assert that $1/e > \epsilon' > 0$, then $1 < x < \infty$. First, we consider the monotonicity of $f(\delta_1') = \frac{a_{\mathrm{lr}}}{2}\delta_1' - d\log(\delta_1')$. Taking the derivative of f shows that $f(\delta_1')$ is monotonically increasing for $\delta_1' \geq 2d/a_{\mathrm{lr}}$. Since $\delta_1' > C_1\log^2(1/\epsilon') = C_1 x^2 \geq C_1 \geq 2d/a_{\mathrm{lr}}$ (note $d \geq 1$), it suffices to establish the claim for $\delta_1' = C_1 x^2$, i.e.,

$$\frac{a_{\mathrm{lr}}}{2}C_1x^2 - d\log\big(C_1x^2\big) \geq x \tag{S77}$$

for $x > 1$. We show that our choice of C_1 satisfies this inequality. First, using the inequality $\log(z) \leq 2(\sqrt{z} - 1)$ for $z > 0$, we can apply this with $z = C_1x^2$ to obtain

$$\frac{a_{\mathrm{lr}}}{2}C_1x^2 - d\log\big(C_1x^2\big) \geq \frac{a_{\mathrm{lr}}}{2}C_1x^2 - 2d\sqrt{C_1}x + 2. \tag{S78}$$

Bounding this trivially because $x^2 > x$ for $x > 1$, we have

$$\frac{a_{\mathrm{lr}}}{2}C_1x^2 - d\log\big(C_1x^2\big) \geq \left(\frac{a_{\mathrm{lr}}}{2}C_1 - 2d\sqrt{C_1}\right)x. \tag{S79}$$

Plugging in our choice of C_1 and simplifying, we have

$$\begin{aligned}
\frac{a_{\mathrm{lr}}}{2}C_1x^2 - d\log\big(C_1x^2\big) &\geq \left(\frac{\left(2d + \sqrt{4d^2 + a_{\mathrm{lr}}}\right)^2}{2a_{\mathrm{lr}}} - 2d\left(\frac{2d + \sqrt{4d^2 + a_{\mathrm{lr}}}}{a_{\mathrm{lr}}}\right)\right)x && \text{(S80a)}\\
&= \left(\frac{8d^2 + 2a_{\mathrm{lr}} + 4d\sqrt{4d^2 + 2a_{\mathrm{lr}}}}{2a_{\mathrm{lr}}} - \frac{8d^2 + 4d\sqrt{4d^2 + 2a_{\mathrm{lr}}}}{2a_{\mathrm{lr}}}\right)x && \text{(S80b)}\\
&= x. && \text{(S80c)}
\end{aligned}$$

Hence, we obtain the desired inequality. □

Lemma 7. *Given $b > 0$, there exists a constant C_2 large enough such that for all $1/e > \epsilon' > 0$ and for all $\delta_1' > C_2 \log^2(1/\epsilon')$,*

$$\frac{2b\delta_1'}{7\log^2(b(\delta_1'+1))} - 22\log(b(\delta_1'+1)) \geq \log\left(\frac{1}{\epsilon'}\right). \tag{S81}$$

Explicitly, such a constant C_2 can be given by

$$C_2 = \max\left(\frac{(18b + (63\cdot 22/2))^3}{b}, 1, \frac{2(7\cdot 16)^2}{b}, \frac{2^3(7\cdot 22\cdot 64)^4}{b}\right). \tag{S82}$$

Proof. For simplicity, throughout this proof, let $x = \log(1/\epsilon')$. Because we assert that $1/e > \epsilon' > 0$, then $1 < x < \infty$. First, we consider the monotonicity of $f(\delta_1') = \frac{2b\delta_1'}{7\log^2(b(\delta_1'+1))} - 22\log(b(\delta_1'+1))$. Taking the derivative of f shows that $f(\delta_1')$ is monotonically increasing for $\delta_1' \geq (18b + (63\cdot 22/2))^3/b$. For $\delta_1' \geq (18b + (63\cdot 22/2))^3/b$, we can make use of $\log(z) \leq 3z^{1/3}, \forall z > 0$ to show that

$$\frac{2b(\delta_1'+1)}{7\log^2(b(\delta_1'+1))} \geq \frac{4}{7}b + 22. \tag{S83}$$

Because $\delta_1' \geq (18b + (63\cdot 22/2))^3/b \geq e/b$, we have

$$\log^3(b(\delta_1'+1)) \geq 1. \tag{S84}$$

Together, we can show that for $\delta_1' \geq (18b + (63\cdot 22/2))^3/b$,

$$f'(\delta_1') = \frac{2b}{7\log^2(b(\delta_1'+1))} - \frac{4b}{7(\delta_1'+1)\log^3(b(\delta_1'+1))} - \frac{22}{\delta_1'+1} \geq 0. \tag{S85}$$

Since $\delta_1' > C_2\log^2(1/\epsilon') = C_2x^2 \geq C_2 \geq (18b + (63\cdot 22/2))^3/b$, it suffices to establish the claim for $\delta_1' = C_2x^2$, i.e.,

$$\frac{2bC_2x^2}{7\log^2(bC_2x^2+b)} - 22\log\big(bC_2x^2+b\big) \geq x \tag{S86}$$

for $x > 1$. We show that our choice of C_2 satisfies this. First, notice that it suffices to show the following two inequalities

$$\frac{bC_2x^2}{7\log^2(bC_2x^2+b)} \geq x \tag{S87}$$

and

$$\frac{bC_2x^2}{7\log^2(bC_2x^2+b)} \geq 22\log\big(bC_2x^2+b\big). \tag{S88}$$

Since $C_2 \geq 1$ and $x > 1$, then $C_2x^2 \geq 1$ and $bC_2x^2 + b \leq 2bC_2x^2$. Then, in Eq. (S87), we have

$$\frac{bC_2x^2}{7\log^2(bC_2x^2+b)} \geq \frac{bC_2x^2}{7\log^2(2bC_2x^2)} \geq \frac{\sqrt{C_2b}}{7\cdot 16\sqrt{2}}x \geq x, \tag{S89}$$

where the second inequality follows using the inequality $\log(z) \leq 4z^{1/4}$ for $z > 0$, applied with $z = 2bC_2x^2$, and the last inequality follows from our choice of C_2. This proves Eq. (S87). Now, to prove Eq. (S88), notice that it suffices to show that

$$\frac{bC_2x^2}{7} \geq 22(4(2bC_2x^2)^{1/4})^3 = 22\cdot 64(2bC_2)^{3/4}x^{3/2}. \tag{S90}$$

This is because, again using the inequality $\log(z) \leq 4z^{1/4}$ with $z = 2bC_2x^2$, then

$$\frac{bC_2x^2}{7} \geq 22\cdot 64(2bC_2)^{3/4}x^{3/2} \geq 22\log^3(2bC_2x^2) \tag{S91}$$

so Eq. (S90) implies Eq. (S88). Thus, it remains to prove Eq. (S90), which is equivalent to

$$C_2 \geq \frac{(7\cdot 22\cdot 64)^4\cdot 2^3}{b}. \tag{S92}$$

Because $x > 1$, our choice of C_2 satisfies the above inequality. □

Lemma 8. *Given $a_{\mathrm{lr}}, b > 0$ and $d \geq 1$, there exists a constant C_3 large enough such that for all $1/e > \epsilon' > 0$ and for all $\delta_1' > C_3 \log^2(1/\epsilon')$,*

$$\frac{2b\delta_1'}{\log^2(b(\delta_1'+1))} - (d+22)\log(b(\delta_1'+1)) \geq \log\left(\frac{1}{\epsilon'}\right) \tag{S93}$$

Explicitly, such a constant C_3 can be given by

$$C_3 = \max\left(\frac{(18b + 63(d+22)/2)^3}{b}, 1, \frac{2(7\cdot 16)^2}{b}, \frac{2^3(7\cdot(d+22)\cdot 64)^4}{b}\right). \tag{S94}$$

Proof. The proof is the same as that of Lemma 7 after replacing 22 by $d + 22$. □

Next, we begin the integral bounds portion of this section and reprove a variant of the lemma introduced in [2].

Lemma 9 (Variant of Lemma 2.5 in [2], Lemma 5 in [1])**.** *For $a > 0$ define*

$$u_a(x) = e^{-a\frac{x}{\log^2(x)}}$$

on the domain $x \in (1,\infty)$, where log *denotes the natural logarithm. For all integers $k \geq 0$ and $t \geq 5504$ such that*

$$a\frac{t}{\log^2 t} > 2k+2, \tag{S95}$$

we have the bound

$$\int_t^{+\infty} x^k u_a(x)\,dx \leq \frac{1}{a\left(1-\frac{2k+2}{\tau(t)}\right)} t^{2k+2} u_a(t), \tag{S96}$$

where $\tau(x) \triangleq ax/\log^2(x)$.

To prove this, we need a bound on the upper incomplete Gamma function:

Lemma 10 (Proposition 2.7 in [8])**.** *Take any real $n \geq 0$. Then,*

$$\Gamma(n+1, z) \leq \frac{1}{1-\frac{n}{z}} z^n e^{-z}, \tag{S97}$$

for all real $z > n$.

Proof of Lemma 9. Define the function

$$\tau(x) \triangleq a\frac{x}{\log^2(x)}. \tag{S98}$$

Here, because we are considering the domain $x \in (1,\infty)$, then this function is well-defined and differentiable. Moreover, it is always positive because $\log^2(x) \geq \log(x) > 0$ for $x > 1$. Also, consider the derivative

$$\frac{d\tau}{dx} = a\left(\frac{\log(x)-2}{\log^3(x)}\right). \tag{S99}$$

Again, this is well-defined because $\log^3(x) > 0$ for $x > 1$. Furthermore, we see that if $x \geq e^2$, then $\frac{d\tau}{dx} > 0$. Thus, for $x \geq e^2$, $\tau(x)$ is monotone increasing. Ultimately, our goal is to bound the integral

$$\int_t^{+\infty} x^k u_a(x)\,dx = \int_t^{+\infty} x^k e^{-\tau(x)}\,dx \tag{S100}$$

by using a substitution $\tau = \tau(x), d\tau = \frac{d\tau}{dx}dx$. Substituting in for x, we use the inverse $x = x(\tau)$ and for the differential dx, we use $dx = \frac{dx}{d\tau}d\tau$ to obtain

$$\int_{\tau(t)}^{+\infty} (x(\tau))^k e^{-\tau}\frac{dx}{d\tau}\,d\tau. \tag{S101}$$

We want to get this into the form of the upper incomplete Gamma function:

$$\Gamma(n+1,z) = \int_{z}^{+\infty} \tau^n e^{-\tau}\, d\tau = n! e^{-z} \sum_{k=0}^{n} \frac{z^k}{k!}. \tag{S102}$$

Thus, we want to find bounds on $\frac{dx}{d\tau}$ and $x(\tau)$ in terms of τ (and constants). Since we define $x(\tau)$ as the inverse of $\tau(x)$, then we know that

$$\frac{dx}{d\tau} = \frac{1}{\frac{d\tau}{dx}} = \frac{1}{a}\left(\frac{\log^3(x)}{\log(x)-2}\right). \tag{S103}$$

We notice here that if $x \geq 28$, then

$$\frac{dx}{d\tau} = \frac{1}{a}\left(\frac{\log^3(x)}{\log(x)-2}\right) \leq \frac{x}{a}. \tag{S104}$$

If we further require $x \geq 5504$, then

$$x \leq \left(\frac{x}{\log^2 x}\right)^2 = \frac{\tau^2}{a^2}. \tag{S105}$$

Using these together, we have that

$$\frac{dx}{d\tau} \leq \frac{x}{a} \leq \frac{\tau^2}{a^3}. \tag{S106}$$

Plugging these into Eq. (S101), we can upper bound our integral

$$\begin{aligned}
\int_{t}^{+\infty} x^k e^{-\tau(x)}\, dx &= \int_{\tau(t)}^{+\infty} (x(\tau))^k e^{-\tau} \frac{dx}{d\tau}\, d\tau && \text{(S107a)}\\
&\leq \int_{\tau(t)}^{+\infty} \frac{\tau^{2k}}{a^{2k}} e^{-\tau} \frac{\tau^2}{a^3}\, d\tau && \text{(S107b)}\\
&= \frac{1}{a^{2k+3}} \int_{\tau(t)}^{+\infty} \tau^{2k+2} e^{-\tau}\, d\tau && \text{(S107c)}\\
&= \frac{1}{a^{2k+3}} \Gamma(2k+3, \tau(t)). && \text{(S107d)}
\end{aligned}$$

Now, applying Lemma 10, we can further bound this:

$$\int_{t}^{+\infty} x^k e^{-\tau(x)}\, dx \leq \frac{1}{a^{2k+3}} \frac{1}{1-\frac{2k+2}{\tau(t)}} (\tau(t))^{2k+2} e^{-\tau(t)} \tag{S108}$$

for $\tau(t) > 2k+2$. Finally, since $\tau(t) \leq at$ for $t \geq e$, then we have

$$\int_{t}^{+\infty} x^k e^{-\tau(x)}\, dx \leq \frac{1}{a\left(1-\frac{2k+2}{\tau(t)}\right)} t^{2k+2} e^{-\tau(t)}. \tag{S109}$$

□

We use this to obtain another integral bound, which is as follows.

Lemma 11. *Let δ_1, ϵ be as in Definition 1. Then, there exists a constant c such that*

$$I = \int_{r=0}^{+\infty} \left(\frac{1}{1-\frac{35\log^2(b(\delta_1+r+1))}{b(\delta_1+r)}}\right) (\delta_1+r+1)^{10} \exp\left(-\frac{2}{7}\frac{b(\delta_1+r)}{\log^2(b(\delta_1+r+1))}\right) dr \leq c\epsilon. \tag{S110}$$

Proof. Using the substitution $x = b(\delta_1+r+1)$, this integral transforms into

$$I = \left(\frac{1}{b}\right)^{n+11} \int_{x=b(\delta_1+1)}^{+\infty} \left(\frac{1}{1-\frac{35\log^2 x}{x-b}}\right) x^{10} \exp\left(-\frac{2}{7}\frac{x-b}{\log^2(x)}\right) dx. \tag{S111}$$

Here, we can show that for our choice of δ_1, $e^{2b/7\log^2(x)}$ and $1/(1-(35\log^2 x)/(x-b))$ are both monotonically decreasing in x. The derivative of the exponential term is

$$\frac{d}{dx}e^{\frac{2}{7}\frac{b}{\log^2 x}} = -\frac{4be^{\frac{2}{7}\frac{b}{\log^2 x}}}{7x\log^3 x}. \tag{S112}$$

To show that the exponential term is monotonically decreasing, we need to show that this derivative is less than 0 for $x \geq b(\delta_1+1)$. We see that $e^{2b/(7\log^2 x)}$ is always nonnegative and $\log^3 x$ is positive as long as $x > 1$ (in which case $x > 0$ as well). Thus, we only require $x > 1$ for this derivative to be less than 0.

Similarly, for the other term, we have the derivative

$$\frac{d}{dx}\frac{1}{1-\frac{35\log^2 x}{x-b}} = -\frac{35\log x(2b-2x+x\log x)}{x(b-x+35\log^2 x)^2}. \tag{S113}$$

In order for this to be less than 0, we see that $(b-x+35\log^2 x)^2$ is always nonnegative and $\log x$ is positive as long as $x > 1$ (in which case $x > 0$ as well). Then, the only term left is $2b-2x+x\log x$, which is positive as long as $\log x > 2(x-b)/x = 2-2b/x$. This is satisfied if $\log x > 2$, which follows when $x > e^2$.

Putting everything together, we see that both of these terms are monotonically decreasing in x for $x > e^2$. In our integral, we have $x \geq b(\delta_1+1)$. However, by our choice of δ_1 in Definition 1, we have that $\delta_1 \geq 5900/b$ so that $b(\delta_1+1) > b\delta_1 \geq 5900 > e^2$. Hence, the condition for these terms to be monotonically decreasing is satisfied for the bounds of the integral.

Thus, because these terms are monotonically decreasing, we can upper bound the integral by

$$I \leq \left(\frac{1}{b}\right)^{11}\exp\left(\frac{2}{7}\frac{b}{\log^2(b(\delta_1+1))}\right)\left(\frac{1}{1-\frac{35\log^2(b(\delta_1+1))}{b\delta_1}}\right)\int_{x=b(\delta_1+1)}^{+\infty} x^{10}e^{-\frac{2}{7}\frac{x}{\log^2(x)}}\,dx. \tag{S114}$$

Now, we can use Lemma 9 to bound this final integral using $k=10, a=2/7$:

$$\int_{x=b(\delta_1+1)}^{+\infty} x^{10}e^{-\frac{2}{7}\frac{x}{\log^2(x)}}\,dx \leq \frac{7}{2}\frac{1}{1-\frac{7(22)\log^2(b(\delta_1+1))}{2b(\delta_1+1)}}(b(\delta_1+1))^{22}\exp\left(-\frac{2}{7}\frac{b(\delta_1+1)}{\log^2(b(\delta_1+1))}\right). \tag{S115}$$

Here, we note that the conditions are satisfied because

$$t = \frac{\gamma(\delta_1+1)}{2v_{\mathrm{lr}}} \geq \frac{\gamma\delta_1}{2v_{\mathrm{lr}}} \geq \max(5900, \alpha, 7(d+11), \theta) \geq 5900, \tag{S116}$$

and it is clear that for $t \geq 5900$ that $at/\log^2 t > 22$. Let $c_1 = 7(1/b)^{11}/2$, and we can combine these bounds:

$$I \leq c_1\exp\left(-\frac{2}{7}\frac{b\delta_1}{\log^2(b(\delta_1+1))}\right)\left(\frac{1}{1-\frac{35\log^2(b(\delta_1+1))}{b\delta_1}}\right)\left(\frac{1}{1-\frac{7(22)\log^2(b(\delta_1+1))}{2b(\delta_1+1)}}\right)(b(\delta_1+1))^{22}. \tag{S117}$$

We can further bound this by

$$I \leq 4c_1\exp\left(\frac{-2\gamma\delta_1+14(22)v_{\mathrm{lr}}\log^3(b(\delta_1+1))}{14v_{\mathrm{lr}}\log^2(b(\delta_1+1))}\right). \tag{S118}$$

Here, this is because of Eq. (S8). This follows because

$$\frac{1}{1-\frac{35\log^2(b(\delta_1+1))}{b\delta_1}} \leq \frac{1}{1-\frac{77\log^2(b(\delta_1+1))}{b(\delta_1+1)}}, \tag{S119}$$

Now, by our choice of δ_1 and Lemma. 7, then we have

$$I \leq 4c_1e^{-\log(1/\epsilon)} = 4c_1\epsilon. \tag{S120}$$

Taking $c = 4c_1$, we arrive at our claim. □

Lemma 12. *Let δ_1, ϵ be as in Definition 1. Then, there exists a constant c' such that*

$$I = \int_{r=0}^{+\infty}\left(\frac{1}{1-\frac{35\log^2(b(\delta_1+r+1))}{b(\delta_1+r)}}\right)(\delta_1+r+1)^{d+10}\exp\left(-\frac{2}{7}\frac{b(\delta_1+r)}{\log^2(b(\delta_1+r+1))}\right)dr \leq c'\epsilon. \tag{S121}$$

Proof. The proof is the same as that of Lemma 11 after replacing x^{10} by x^{d+10}. Moreover, in the final steps, instead of using Eq. (S8) and Lemma 7, we use Eq. (S9) and Lemma 8, respectively. □

II. NORM INEQUALITY FOR OBSERVABLES

The efficiency of learning depends strongly on the complexity of the target functions we would like to learn. One way to characterize the complexity of the target function is to consider an appropriate norm of the function. Given an observable $O = \sum_P \alpha_P P$ specified by the Pauli coefficients α_P, Theorem 3 shows that having a smaller ℓ_1-norm $\sum_P |\alpha_P|$ on the Pauli coefficients implies that the ground state property $\mathrm{Tr}(O\rho(x))$ can be better approximated by a simple function. This motivates the derivation of bounds on $\sum_P |\alpha_P|$.

A technical contribution of this work is to develop a norm inequality relating the ℓ_1-norm of the Pauli coefficients $\sum_P |\alpha_P|$ to the spectral norm $\|O\|_\infty$ (the largest singular value). To state this result precisely, we first present some formal definitions. Throughout the remainder of this section, we consider labelling the n qubits in a d-dimensional lattice with a d-tuple, $\ell = (\ell_1, \ldots, \ell_d)$, where each $\ell_k \in \{1, \ldots, \lfloor \sqrt[d]{n} \rfloor\}$.

Definition 4 (Domain of an observable). *Let O be an arbitrary observable in a finite d-dimensional space. Then, define the* domain $\mathsf{dom}(O) \subseteq \{1, \ldots, \lfloor \sqrt[d]{n} \rfloor\}^d$ *of O to be the set of qubits that O acts nontrivially on.*

Definition 5 (geometrically local with range R). *Let O be an arbitrary observable in a finite d-dimensional space and let $\mathsf{dom}(O) \subseteq \{1, \ldots, \lfloor \sqrt[d]{n} \rfloor\}^d$ be its domain. Moreover, let $\mathsf{dom}(O)_k = \pi_k(\mathsf{dom}(O)) \subseteq \{1, \ldots, \lfloor \sqrt[d]{n} \rfloor\}$, where $\pi_k : \mathbb{Z}^d \to \mathbb{Z}$ is the projection map onto the kth coordinate. Let $R_{O,k} \triangleq \max(\mathsf{dom}(O)_k) - \min(\mathsf{dom}(O)_k)$. The observable O is* geometrically local with range R *if $R_{O,k} \leq R_k$, for all $k = 1, \ldots, d$ and*

$$R \triangleq \prod_{k=1}^{d} R_k. \tag{S1}$$

In cases when the range $R = \mathcal{O}(1)$ is unimportant, we simply say that O is geometrically local.

We can now properly state the norm inequality relating the Pauli-1 norm to the spectral norm.

Theorem 4 (Detailed restatement of Theorem 2). *Given an observable $O = \sum_P \alpha_P P$ that can be written as a sum of geometrically local observables with range R in a finite d-dimensional space, we have*

$$\sum_P |\alpha_P| \leq 2^d R \cdot 4^R \|O\|_\infty. \tag{S2}$$

If we additionally require that $\|O\|_\infty = \mathcal{O}(1)$, we have the following corollary.

Corollary 4. *Given an observable $O = \sum_P \alpha_P P$ with $\|O\|_\infty = \mathcal{O}(1)$ that can be written as a sum of geometrically local observables in a finite d-dimensional space with $R = \mathcal{O}(1)$, we have $\sum_P |\alpha_P| = \mathcal{O}(1)$.*

In order to establish the above norm inequality, we consider an explicit algorithm for constructing a state ρ satisfying $\sum_Q |\alpha_Q| \leq C\,\mathrm{Tr}(O\rho)$. In this way, bounding $\mathrm{Tr}(O\rho)$ above by $\|O\|_\infty$ gives the desired inequality. We briefly discuss the idea of the algorithm. First, we consider the set of all geometrically local blocks over the n qubits. Then, we consider all Pauli observables Q with nonzero α_Q and the qubits that Q acts on. For each block, if the qubits that Q acts on are all inside that block, we put Q inside of this block. If there are multiple such blocks, we choose an arbitrary one to put Q in so that each Pauli observable Q is in exactly one block. After that, we separate all blocks into a few disjoint layers of blocks. Each layer contains many blocks that are sufficiently far from one another, and each block contains some Pauli observables. We select the layer that has the largest $\sum_Q |\alpha_Q|$, where this sum is over all Pauli observables inside that layer. To construct the state ρ, we let ρ be the maximally mixed state on qubits outside of the selected layer. For each block in the selected layer, we choose ρ to be a state that maximizes the sum of the Pauli terms in the block. With a careful analysis, the constructed state ρ satisfies the desired norm inequality.

A. Facts and lemmas

Before proving Theorem 4, we give a few definitions, facts and lemmas.

Definition 6 (geometrically local Pauli observables). *Throughout this section, we consider $S^{(\mathrm{geo})}$ to be the set of all geometrically local Pauli observables with a constant range $R = \mathcal{O}(1)$.*

The following fact can be easily shown by considering the Pauli decomposition of each geometrically local observable in the sum.

Fact 1. *Any observable O that can be written as a sum of geometrically local observables can also be written as a sum of geometrically local Pauli observables. Thus, we can write $O = \sum_P \alpha_P P$, where $\alpha_P = 0$ for all $P \notin S^{(\mathrm{geo})}$.*

A construction of the mixed state that we are going to use throughout the proof is the following. The key idea is that $|\mathrm{Tr}(P_i\rho)| = 1/k$ for $i = 1, \ldots, k$, and $|\mathrm{Tr}(P\rho)| = 0$ for any $P \in \{I, X, Y, Z\}^{\otimes n} \setminus \{I, P_1, \ldots, P_k\}$.

Lemma 13. *Let $P_1, \ldots, P_k \in \{I, X, Y, Z\}^{\otimes n}$. Suppose that $P_i \neq I^{\otimes n}$ for all $i = 1, \ldots, k$. Then*

$$\rho = \frac{I + \frac{\pm P_1 \pm \cdots \pm P_k}{k}}{2^n} \tag{S3}$$

is a density operator, i.e., it is positive semidefinite and has unit trace.

Proof. First, we can easily show that ρ has unit trace. Let $P_i = \bigotimes_{j=1}^n P_{i,j}$ for all $i = 1, \ldots, k$, where $P_{i,j} \in \{I, X, Y, Z\}$. Then, we have

$$\mathrm{Tr}(\rho) = \frac{1}{2^n}\left(\mathrm{Tr}(I) \pm \frac{1}{k}(\mathrm{Tr}(P_1) \pm \cdots \pm \mathrm{Tr}(P_k))\right) \tag{S4a}$$

$$= \frac{1}{2^n}\left(2^n \pm \frac{1}{k}\left(\prod_{j=1}^n \mathrm{Tr}(P_{1,j}) \pm \cdots \pm \prod_{j=1}^n \mathrm{Tr}(P_{k,j})\right)\right) \tag{S4b}$$

$$= 1, \tag{S4c}$$

where the last equality follows because the trace of a nonidentity Pauli matrix is 0, and we assume that $P_i \neq I^{\otimes n}$ so that the $P_{i,j}$ are not all identity. To show that ρ is positive semidefinite, it suffices to prove that the eigenvalues of $(\pm P_1 \pm \cdots \pm P_k)/k$ are between -1 and 1. Then, when this is summed with the identity matrix which has eigenvalue $+1$, the eigenvalues are nonnegative. We see this using the spectral norm

$$\left\| \frac{\pm P_1 \pm \cdots \pm P_k}{k} \right\|_\infty \leq \frac{1}{k}\left(\|P_1\|_\infty + \cdots + \|P_k\|_\infty\right) = 1, \tag{S5}$$

which concludes our proof. □

Now, we want to define an operation that is useful throughout the proof.

Definition 7 (Restriction of a Pauli operator)**.** *Let $P \in \{I, X, Y, Z\}^{\otimes n}$. Write $P = \bigotimes_{\ell \in \{1, \ldots, \lfloor \sqrt[d]{n} \rfloor\}^d} P_\ell$ for $P_\ell \in \{I, X, Y, Z\}$. Let $S \subseteq \{1, \ldots, \lfloor \sqrt[d]{n} \rfloor\}^d$ be a subset of qubits. The* restriction of P to the subset of qubits S *is the substring of Paulis that act on S:*

$$\mathsf{restrict}(P; S) \triangleq P_S \triangleq \bigotimes_{\ell \in S} P_\ell. \tag{S6}$$

In Definition 7, the subscript notation is used to be consistent with the more standard notation of P_k to denote a Pauli acting on qubit k.

B. Proof of Theorem 4

The key idea is to upper bound $\sum_P |\alpha_P|$ by a constant times $\mathrm{Tr}(O\rho)$ for some test state ρ. We construct such a ρ with a similar form to that seen in Lemma 13. Then, because ρ is positive semidefinite and has unit trace by Lemma 13, $\mathrm{Tr}(O\rho) \leq \|O\|_\infty$. Putting everything together, we have

$$\sum_P |\alpha_P| \leq 2^d R \cdot 4^R \,\mathrm{Tr}(O\rho) \leq 2^d R \cdot 4^R \|O\|_\infty, \tag{S7}$$

as required. Thus, it suffices to consider this intermediate step of finding a quantum state ρ such that $2^d R \cdot 4^R \,\mathrm{Tr}(O\rho) \geq \sum_P |\alpha_P|$. To this end, we consider dividing our space of all Pauli observables into different sets and focus on one set, which educates our choice of ρ.

Consider some Pauli observable $P \in S^{(\text{geo})}$, where $S^{(\text{geo})}$ is the set of all geometrically local Pauli observables. Since P is geometrically local, by Definition 5, there exist constants R_k for $k = 1, \ldots, d$ that serve as the maximum range of qubits that a Pauli observable covers in the kth dimension. We want to divide our d-dimensional space into *blocks* of R_k qubits in each dimension. These blocks of qubits are

$$B_{(\vec{i},\vec{j})} \triangleq \{\text{qubits } \ \ell = (\ell_1, \ldots, \ell_d) : \ell_k \in [(2i_k - 2)R_k + j_k + 1, (2i_k - 1)R_k + j_k], \ \ \forall k \in \{1, \ldots, d\}\}, \tag{S8}$$

where $\vec{i} = (i_1, \ldots, i_d)$ and $\vec{j} = (j_1, \ldots, j_d)$. We construct these blocks for $i_k = 1, \ldots, \lfloor \frac{\lfloor \sqrt[d]{n} \rfloor - j_k + R_k}{2R_k} \rfloor$ and $j_k = 0, \ldots, 2R_k - 1$ for $k = 1, \ldots, d$. Here, we are dividing the d-dimensional space into blocks of $R = \prod_{k=1}^d R_k$ qubits, where each block is index by $\vec{i}$ and is separated from the next by R_k qubits in the kth dimension. We refer to this gap between the blocks as the *buffer*. Denote the buffer as

$$B'_{\vec{j}} \triangleq \{1, \ldots, \lfloor \sqrt[d]{[n]} \rfloor\}^d \setminus \left(\bigcup_{\vec{i}} B_{(\vec{i},\vec{j})} \right), \tag{S9}$$

where the union is over all possible vectors $\vec{i}$ such that i_k ranges from 1 to $\lfloor \frac{\lfloor \sqrt[d]{n} \rfloor - j_k + R_k}{2R_k} \rfloor$. This separation using the buffer region is so that no Pauli term can act on qubits in two blocks at once, which we use later. Moreover, we are considering possible shifts of these blocks by j_k qubits in each of the dimensions. Notice that there are only $2R_k$ possible shifts in each dimension until the blocks align with the original positioning of another block. Consider the related set consisting of the Pauli terms that act only on qubits in a given block

$$S_{(\vec{i},\vec{j})} \triangleq \{P : \text{for all qubits } k \in \mathsf{dom}(P), \text{ then } k \in B_{(\vec{i},\vec{j})}\} \setminus \left(\bigcup_{(\vec{i}',\vec{j}') \leq (\vec{i},\vec{j})} S_{(\vec{i}',\vec{j}')} \right), \tag{S10}$$

where we define $(\vec{i}', \vec{j}') \leq (\vec{i}, \vec{j})$ using the standard lexicographical order, i.e.,

$$(\vec{i}', \vec{j}') = ((i'_1, \ldots, i'_d), (j'_1, \ldots, j'_d)) \leq (\vec{i}, \vec{j}) = ((i_1, \ldots, i_d), (j_1, \ldots, j_d)) \tag{S11}$$

if and only if $\vec{i}' < \vec{i}$, or $\vec{i}' = \vec{i}$ and $\vec{j}' \leq \vec{j}$. Here, $\vec{i}' \leq \vec{i}$ if and only if $i'_1 < i_1$, or $i'_1 = i_1$ and $i'_2 < i_2$, or, etc. Thus, we create these sets $S_{(i,j)}$ sequentially according to this ordering. We remove previous sets so that each $S_{(\vec{i},\vec{j})}$ is disjoint from other sets $S_{(\vec{i}',\vec{j}')}$.

Now, taking a union over all $\vec{i}$, we can consider the Pauli terms acting on these blocks together. The resulting sets then only differ based on the shift of j_k qubits in each dimension.

$$U_{\vec{j}} \triangleq \bigcup_{\vec{i}} S_{(\vec{i},\vec{j})}. \tag{S12}$$

Figure S3 illustrates all these definitions. We now consider $\sum_{P \in U_{\vec{j}}} |\alpha_P|$, where these α_P are the coefficients in $O = \sum_P \alpha_P P$. We want to pick the set $U_{\vec{j}}$ such that this sum is largest, i.e.

$$\vec{j}^* \triangleq \underset{\substack{0 \leq j_1 \leq 2R_1 - 1 \\ \vdots \\ 0 \leq j_d \leq 2R_d - 1}}{\arg\max} \sum_{P \in U_{\vec{j}}} |\alpha_P|. \tag{S13}$$

We now focus on the set $U_{\vec{j}^*}$. To justify this choice, we can think of each of the sets shifted by $\vec{j}$ as breaking up the sum $\sum_P |\alpha_P|$ into different disjoint sums. This is a result of our earlier choice for $U_{\vec{j}}$ to contain a disjoint set of Pauli terms. Then, the maximum over all shifts $\vec{j}$ of $\sum_{P \in U_{\vec{j}}} |\alpha_P|$ is greater than the average over all shifts. In other words, we have

$$\sum_{P \in U_{\vec{j}^*}} |\alpha_P| \geq \frac{1}{2^d R} \sum_P |\alpha_P|, \tag{S14}$$

where recall that $R = \prod_{k=1}^d R_k$. Relating back to our original goal, it remains to find a test state ρ such that

$$\mathrm{Tr}(O\rho) \geq \frac{1}{4^R} \sum_{P \in U_{\vec{j}^*}} |\alpha_P|. \tag{S15}$$

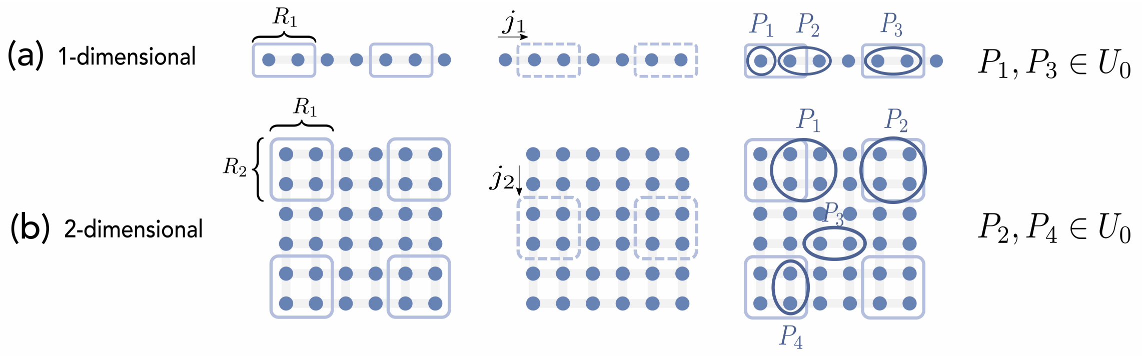

Figure S3: **Intuition behind proof construction of Theorem 4 for the cases of $d = 1$ (a) and $d = 2$ (b).** In both cases, the idea is to divide our qubits (blue circles) in d-dimensional space into blocks (light blue boxes), and consider the quantity we wish to bound in these blocks. Note that all qubits not highlighted are in the buffer region. The first column in the figure depicts the unshifted blocks, i.e., $\vec{j} = 0$. The second column displays an example of shifted blocks (dashed boxes). Finally, the last column considers Pauli terms (dark blue circles) acting on the qubits circled and indicates if they are contained in U_0, defined in Eq. (S12).

Once we have this, we can conclude that

$$2^d R \cdot 4^R \|O\|_\infty \geq 2^d R \cdot 4^R \operatorname{Tr}(O\rho) \geq 2^d R \sum_{P \in U_{\vec{j}^*}} |\alpha_P| \geq \sum_P |\alpha_P|, \tag{S16}$$

proving our claim, where the first inequality follows because ρ is positive semidefinite and has unit trace from Lemma 13. In what follows, we aim to define this ρ based on the set $U_{\vec{j}^*}$ and show that this inequality holds.

The idea is to have ρ as the maximally mixed state on qubits in the buffer region $B'_{\vec{j}^*}$ and be a state of the form in Lemma 13 for qubits in $\bigcup_{\vec{i}} B_{(\vec{i},\vec{j}^*)}$. In this way, when we take $\operatorname{Tr}(O\rho)$, any Pauli terms not in $U_{\vec{j}^*}$ contribute 0 while Pauli terms P in $U_{\vec{j}^*}$ contribute a constant times $|\alpha_P|$. Explicitly, we define ρ as

$$\rho \triangleq \bigotimes_{\vec{i}} \left(\frac{1}{2^R} \left(I_{B_{(\vec{i},\vec{j}^*)}} + \frac{1}{|S_{(\vec{i},\vec{j}^*)}|} \sum_{Q \in S_{(\vec{i},\vec{j}^*)}} (-1)^{\delta_{\{\alpha_Q < 0\}}} Q_{B_{(\vec{i},\vec{j}^*)}} \right) \right) \bigotimes_{\ell \in B'_{\vec{j}^*}} \frac{I_\ell}{2} \triangleq \bigotimes_{\vec{i}} \rho_{\vec{i}} \bigotimes_{\ell \in B'_{\vec{j}^*}} \frac{I_\ell}{2}, \tag{S17}$$

where $\delta_{\{\alpha_Q < 0\}}$ is 1 when $\alpha_Q < 0$ and 0 otherwise. Also, the tensor product is again over all possible vectors $\vec{i}$ such that each entry i_k ranges from 1 to $\lfloor \frac{\lfloor \sqrt[d]{n} \rfloor - j_k + R_k}{2R_k} \rfloor$. Here, we are using the notation from Definition 7 to denote quantum operations restricted to their action on a given set of qubits. By Lemma 13, $\rho_{\vec{i}}$ is a proper quantum state that is positive semidefinite and has unit trace; hence ρ is a quantum state. Now, we want to calculate $\operatorname{Tr}(O\rho)$. Recall that $O = \sum_P \alpha_P P$. Taking the trace, we have

$$\operatorname{Tr}(O\rho) = \sum_P \alpha_P \operatorname{Tr}(P\rho). \tag{S18}$$

There are four cases that can occur regarding $\mathsf{dom}(P)$.

1. P acts nontrivially on some qubits in the buffer region, i.e., $\mathsf{dom}(P) \subseteq B_{(\vec{i}_P,\vec{j}^*)} \cup B'_{\vec{j}^*}$ for some $\vec{i}_P$.

2. P acts trivially on all qubits in the buffer region, but P acts nontrivially on qubits in two or more blocks, i.e., $\mathsf{dom}(P) \subseteq B_{(\vec{i}_{P,1},\vec{j}^*)} \cup B_{(\vec{i}_{P,2},\vec{j}^*)}$ for some $\vec{i}_{P,1}, \vec{i}_{P,2}$.

3. P acts nontrivially only on qubits in a single block but P is not in the set $U_{\vec{j}^*}$, i.e., $\mathsf{dom}(P) \subseteq B_{(\vec{i}_P,\vec{j}^*)}$ for some $\vec{i}_P$, but $P \notin S_{(\vec{i}_P,\vec{j}^*)}$.

4. P acts nontrivially only on qubits in a single block and P is in the set U_{j^*}, i.e., $\mathsf{dom}(P) \subseteq B_{(\vec{i}_P,\vec{j}^*)}$ for some $\vec{i}_P$ and $P \in S_{(\vec{i}_P,\vec{j}^*)}$.

We compute $\mathrm{Tr}(P\rho)$ for each of these cases. We note that $\mathrm{Tr}\big(\rho_{\vec{i}}\big) = 1$ in all these calculations.

For Case 1, it suffices to consider the case where P acts on only one qubit in the buffer region, i.e. there exists a qubit $\ell^* \in B'_{\vec{j}^*}$ such that $\ell^* \in \mathsf{dom}(P)$ and $\mathsf{dom}(P) \setminus \{\ell^*\} \subseteq B_{(\vec{i}_P,\vec{j}^*)}$. Then, the state $P\rho$ is as follows

$$P\rho = \left(\bigotimes_{\vec{i}\neq\vec{i}_P} \rho_{\vec{i}} \bigotimes_{\substack{\ell\in B'_{\vec{j}^*}\\ \ell\neq\ell^*}} \frac{I_\ell}{2} \right) \tag{S19a}$$

$$\otimes \left(\frac{1}{2^R} \left(P_{B_{(\vec{i}_P,\vec{j}^*)}} + \frac{1}{|S_{(\vec{i}_P,\vec{j}^*)}|} \sum_{Q\in S_{(\vec{i}_P,\vec{j}^*)}} (-1)^{\delta_{\{\alpha_Q<0\}}} P_{B_{(\vec{i}_P,\vec{j}^*)}} Q_{B_{(\vec{i}_P,\vec{j}^*)}} \right) \otimes \frac{P_{\ell^*}}{2} \right), \tag{S19b}$$

where we are again using the notation from Definition 7. Taking the trace of this state, since the trace of $I/2$ is 1, we have

$$\mathrm{Tr}(P\rho) = \frac{\mathrm{Tr}\left(\frac{P_{\ell^*}}{2}\right)}{2^R} \left(\mathrm{Tr}\Big(P_{(B_{(\vec{i}_P,\vec{j}^*)})}\Big) \right. \tag{S20a}$$

$$\left. + \frac{1}{|S_{(\vec{i}_P,\vec{j}^*)}|} \sum_{Q\in S_{(\vec{i}_P,\vec{j}^*)}} (-1)^{\delta_{\{\alpha_Q<0\}}} \mathrm{Tr}\Big(P_{B_{(\vec{i}_P,\vec{j}^*)}} Q_{B_{(\vec{i}_P,\vec{j}^*)}} \Big) \right) \tag{S20b}$$

$$= 0, \tag{S20c}$$

where the last equality follows because the trace of a nonidentity Pauli string is 0 and

$$\mathrm{Tr}\Big(P_{B_{(\vec{i}_P,\vec{j}^*)}} Q_{B_{(\vec{i}_P,\vec{j}^*)}} \Big) = 2^R \delta_{\{P_{B_{(\vec{i}_P,\vec{j}^*)}} = Q_{B_{(\vec{i}_P,\vec{j}^*)}}\}}. \tag{S21}$$

Here, $P_{B_{(\vec{i}_P,\vec{j}^*)}} \neq Q_{B_{(\vec{i}_P,\vec{j}^*)}}$ because $Q \in S_{(\vec{i}_P,\vec{j}^*)}$ so that Q acts nontrivially only on qubits in $B_{(\vec{i}_P,\vec{j}^*)}$ while P acts nontrivially on $\ell^* \notin B_{(\vec{i}_P,\vec{j}^*)}$. Thus, Case 1 contributes 0 to $\mathrm{Tr}(O\rho)$.

Next, we consider Case 2. In Case 2, we consider what happens if P acts nontrivially on qubits in more than one block, i.e., $\mathsf{dom}(P) \subseteq B_{(\vec{i}_{P,1},\vec{j}^*)} \cup B_{(\vec{i}_{P,2},\vec{j}^*)}$. However, this case is in fact not possible by construction because the buffer region between $B_{(\vec{i}_{P,1},\vec{j}^*)}$ and $B_{(\vec{i}_{P,2},\vec{j}^*)}$ is of size R_k in each of the dimensions. Recall that R_k is the largest distance between two qubits that any P acts on in the kth dimension. Thus, it is not possible for P to span across the buffer region, so this case cannot occur. Hence, it trivially contributes 0 to $\mathrm{Tr}(O\rho)$.

Now, we consider Case 3. From the previous two cases, we see that P can only act nontrivially on qubits in a single block $B_{(\vec{i}_P,\vec{j}^*)}$ to contribute to $\mathrm{Tr}(O\rho)$. However, by construction of the sets $S_{(\vec{i},\vec{j})}$, in order to make them disjoint, it is possible that $P \notin S_{(\vec{i}_P,\vec{j}^*)}$ despite it acting on the correct block of qubits. We show that this also contributes 0 to $\mathrm{Tr}(O\rho)$. Taking the trace, we have

$$\mathrm{Tr}(P\rho) = \frac{1}{2^R} \left(\mathrm{Tr}\Big(P_{(B_{(\vec{i}_P,\vec{j}^*)})}\Big) + \frac{1}{|S_{(\vec{i}_P,\vec{j}^*)}|} \sum_{Q\in S_{(\vec{i}_P,\vec{j}^*)}} (-1)^{\delta_{\{\alpha_Q<0\}}} \mathrm{Tr}\Big(P_{B_{(\vec{i}_P,\vec{j}^*)}} Q_{B_{(\vec{i}_P,\vec{j}^*)}} \Big) \right) = 0, \tag{S22}$$

where the last equality follows because the trace of a nonidentity Pauli string is 0 and

$$\mathrm{Tr}\Big(P_{B_{(\vec{i}_P,\vec{j}^*)}} Q_{B_{(\vec{i}_P,\vec{j}^*)}} \Big) = 2^R \delta_{\{P_{B_{(\vec{i}_P,\vec{j}^*)}} = Q_{B_{(\vec{i}_P,\vec{j}^*)}}\}}. \tag{S23}$$

Here, $P_{B_{(\vec{i}_P,\vec{j}^*)}} \neq Q_{B_{(\vec{i}_P,\vec{j}^*)}}$ because $Q \in S_{(\vec{i}_P,\vec{j}^*)}$ while we know from this case that $P \notin S_{(\vec{i}_P,\vec{j}^*)}$.

Finally, we consider Case 4. From the previous cases, we see that the only remaining possibility is that P acts nontrivially on qubits in a single block $B_{(\vec{i}_P,\vec{j}^*)}$ and is also contained in a set $S_{(\vec{i}_P,\vec{j}^*)}$. Computing the trace, we have

$$\mathrm{Tr}(P\rho) = \frac{1}{2^R} \left(\mathrm{Tr}\Big(P_{(B_{(\vec{i}_P,\vec{j}^*)})}\Big) + \frac{1}{|S_{(\vec{i}_P,\vec{j}^*)}|} \sum_{Q\in S_{(\vec{i}_P,\vec{j}^*)}} (-1)^{\delta_{\{\alpha_Q<0\}}} \mathrm{Tr}\Big(P_{B_{(\vec{i}_P,\vec{j}^*)}} Q_{B_{(\vec{i}_P,\vec{j}^*)}} \Big) \right) \tag{S24a}$$

$$= \frac{1}{2^R} \frac{1}{|S_{(\vec{i}_P,\vec{j}^*)}|} \sum_{Q \in S_{(\vec{i}_P,\vec{j}^*)}} (-1)^{\delta_{\{\alpha_Q<0\}}} \operatorname{Tr}\Big(P_{B_{(\vec{i}_P,\vec{j}^*)}} Q_{B_{(\vec{i}_P,\vec{j}^*)}}\Big) \tag{S24b}$$

$$= \frac{1}{|S_{(\vec{i}_P,\vec{j}^*)}|} (-1)^{\delta_{\{\alpha_P<0\}}}. \tag{S24c}$$

Here, we are using that the trace of a nonidentity Pauli string is 0 and $\operatorname{Tr}\Big(P_{B_{(\vec{i}_P,\vec{j}^*)}} Q_{B_{(\vec{i}_P,\vec{j}^*)}}\Big) = 2^R \delta_{\{P_{B_{(\vec{i}_P,\vec{j}^*)}} = Q_{B_{(\vec{i}_P,\vec{j}^*)}}\}}$. Because $P \in S_{(\vec{i}_P,\vec{j}^*)}$, there exists a $Q \in S_{(\vec{i}_P,\vec{j}^*)}$ such that $P = Q$ so that the sum over $S_{(\vec{i}_P,\vec{j}^*)}$ then collapses to this P. Thus, for this case, P contributes a nonzero amount to $\operatorname{Tr}(O\rho)$. Summing over all $P \in U_{\vec{j}^*}$, we have a total contribution of

$$\sum_{P \in U_{\vec{j}^*}} \alpha_P \operatorname{Tr}(P\rho) = \sum_{P \in U_{\vec{j}^*}} \frac{1}{|S_{(\vec{i}_P,\vec{j}^*)}|} (-1)^{\delta_{\{\alpha_P<0\}}} \alpha_P = \sum_{P \in U_{\vec{j}^*}} \frac{1}{|S_{(\vec{i}_P,\vec{j}^*)}|} |\alpha_P|. \tag{S25}$$

Thus, putting everything together, only Case 4 contributed a nonzero amount to $\operatorname{Tr}(O\rho)$, so we have

$$\operatorname{Tr}(O\rho) = \sum_{P \in U_{\vec{j}^*}} \frac{1}{|S_{(\vec{i}_P,\vec{j}^*)}|} |\alpha_P|. \tag{S26}$$

Here, we know that

$$|S_{(\vec{i}_P,\vec{j}^*)}| \leq 4^R \tag{S27}$$

because $|B_{(\vec{i}_P,\vec{j}^*)}| = R$ and on $R = \prod_k R_k$ qubits, there are 4^R possible Pauli terms (i.e., I, X, Y, Z on each of the qubits). Then, we have

$$\operatorname{Tr}(O\rho) \geq \frac{1}{4^R} \sum_{P \in U_{\vec{j}^*}} |\alpha_P|. \tag{S28}$$

As explained previously, this suffices to conclude the proof. □

III. ML ALGORITHM AND SAMPLE COMPLEXITY

In this section, we present our machine learning algorithm and prove that it can approximate $\operatorname{Tr}(O\rho(x))$ given training data size N scaling logarithmically in system size n. To do so, we leverage the results in Section I and Section II heavily.

Recall that we consider an unknown family of n-qubit geometrically local Hamiltonians $\{H(x) : x \in [-1,1]^m\}$ in a finite d-dimensional space such that $H(x) = \sum_{j=1}^L h_j(\vec{x}_j)$, where $\vec{x}_j \in \mathbb{R}^q, q = \mathcal{O}(1)$, and x is the concatenation of the L vectors $\vec{x}_1, \ldots, \vec{x}_L$. We also assume that the spectral gap of $H(x)$ is lower bounded by a constant γ over $[-1,1]^m$ and $\rho(x)$ is the ground state of $H(x)$. We also consider an unknown observable O with $\|O\|_\infty = \mathcal{O}(1)$ that can be written as a sum of geometrically local observables and an arbitrary unknown distribution $\mathcal{D}$ over $[-1,1]^m$.

In what follows, we first present a full description of the proposed ML algorithm in Section III A. In Section III B, we then state the rigorous guarantee achieved by this ML algorithm. Next, we find a bound required to utilize ℓ_1-regularized regression in Section III C. Then, we bound the in-sample error on the training data in Section III D by showing that the function $g(x)$ for approximating $\operatorname{Tr}(O\rho(x))$ as defined in Eq. (S60) of Section I achieves small training error. Finally, we use standard results in machine learning theory to bound the prediction error in Section III E.

A. ML algorithm

This section is dedicated to describing the ML algorithm in detail. Let $1/e > \epsilon_1, \epsilon_2, \epsilon_3 > 0$. The ML algorithm is also given training data $\{(x_\ell, y_\ell)\}_{\ell=1}^N$ consisting of parameters x_ℓ sampled from an arbitrary unknown distribution $\mathcal{D}$ over $[-1,1]^m$ along with an estimator y_ℓ of $\operatorname{Tr}(O\rho(x_\ell))$ such that $|y_\ell - \operatorname{Tr}(O\rho(x_\ell))| \leq \epsilon_2$.

Given this, we first redefine several notions from Section I in terms of ϵ_1. We utilize these definitions in the remainder of Section III. We begin by redefining δ_1 and I_P, originally defined in Def. 1, 2, respectively. Define δ_1 as

$$\delta_1 \triangleq \max\left(C_{\max}\log^2(2C/\epsilon_1), C_4, C_5, \frac{\max(5900, \alpha, 7(d+11), \theta)}{b}\right), \tag{S1}$$

where all constants $b, v_{\rm lr}, C_{\max}, C_4, C_5, \alpha, \theta$ are defined as in Def. 1 and C is defined in Lemma 2. Using this definition of δ_1, let I_P be defined as

$$I_P \triangleq \{c \in \{1, \ldots, m\} : d_{\rm obs}(h_{j(c)}, P) \leq \delta_1\}, \tag{S2}$$

as in Eq. (S11). Now, we can redefine the quantities from Def. 3 used to approximate the ground state property as a sum of discretized functions. Let δ_2 be given by

$$\delta_2 \triangleq \frac{1}{\left\lceil \frac{2\sqrt{C'|I_P|}}{\epsilon_1} \right\rceil}, \tag{S3}$$

where C' is defined in Lemma 2. From this, we can define the discretized parameter space X_P, which contains parameter vectors that are 0 outside of I_P and take on discrete values inside of I_P:

$$X_P \triangleq \left\{ \begin{array}{ll} x \in [-1,1]^m : \text{if } c \notin I_P, x_c = 0 \\ \qquad\qquad\qquad\;\; \text{if } c \in I_P, x_c \in \{0, \pm\delta_2, \pm 2\delta_2, \ldots, \pm 1\} \end{array} \right\}. \tag{S4}$$

Furthermore, for each discretized vector $x' \in X_P$, let $T_{x,P}$ be the set of vectors close to x' for coordinates in I_P:

$$T_{x,P} \triangleq \left\{ x' \in [-1,1]^m : -\frac{\delta_2}{2} < x_c - x'_c \leq \frac{\delta_2}{2}, \ \forall c \in I_P \right\}. \tag{S5}$$

Finally, we define an additional hyperparameter $B > 0$ as

$$B \triangleq 2^{\mathcal{O}(\mathrm{polylog}(1/\epsilon_1))}. \tag{S6}$$

With these definitions in place, we can discuss the ML algorithm. At a high level, the algorithm first maps the parameter space into a high-dimensional feature space. Then, the ML algorithm learns a linear function in this feature space using ℓ_1-regularized regression.

In particular, the feature map ϕ maps $x \mapsto \phi(x)$, where $x \in [-1,1]^m$ is an m-dimensional vector while $\phi(x) \in \mathbb{R}^{m_\phi}$ is an m_ϕ-dimensional vector with

$$m_\phi \triangleq \sum_{P \in S^{\rm geo}} |X_P|. \tag{S7}$$

Here, $S^{(\rm geo)}$ denotes the set of all geometrically local Pauli observables as in Def. 6. Each coordinate of $\phi(x)$ is indexed by $x' \in X_P, P \in S^{(\rm geo)}$ and is defined as

$$\phi(x)_{x',P} \triangleq \mathbb{1}[x \in T_{x',P}]. \tag{S8}$$

The hypothesis class for our proposed ML algorithm consists of linear functions in this feature space, i.e., functions of the form $h(x) = \mathbf{w} \cdot \phi(x)$. The classical ML model learns such a function using ℓ_1-regularized regression (LASSO) [9–11] over the feature space. Namely, given the hyperparameter $B > 0$ defined above, we utilize LASSO to find an m_ϕ-dimensional vector $\mathbf{w}^*$ from the following optimization problem that minimizes the training error $\frac{1}{N}\sum_{\ell=1}^N |\mathbf{w} \cdot \phi(x_\ell) - y_\ell|^2$,

$$\min_{\substack{\mathbf{w} \in \mathbb{R}^{m_\phi} \\ \|\mathbf{w}\|_1 \leq B}} \frac{1}{N}\sum_{\ell=1}^N |\mathbf{w} \cdot \phi(x_\ell) - y_\ell|^2, \tag{S9}$$

where y_ℓ approximates $\mathrm{Tr}(O\rho(x_\ell))$. We denote the learned function by $h^*(x) = \mathbf{w}^* \cdot \phi(x)$. Importantly, this learned function does not need to achieve the minimum training error. In the following, we consider the vector $\mathbf{w}^*$ to yield a training error that is larger than the minimum training error by at most $\epsilon_3/2$.

B. Rigorous guarantee

Given these definitions and the ML algorithm, we prove the following theorem. The theorem stated in the main text corresponds to $\epsilon_1 = 0.2\epsilon$, $\epsilon_2 = \epsilon$, and $\epsilon_3 = 0.4\epsilon$. Hence $(\epsilon_1 + \epsilon_2)^2 \leq 1.44\epsilon^2 \leq 0.53\epsilon$ and $(\epsilon_1 + \epsilon_2)^2 + \epsilon_3 \leq \epsilon$.

Theorem 5. *Let $1/e > \epsilon_1, \epsilon_2, \epsilon_3 > 0$ and $\delta > 0$. Given training data $\{(x_\ell, y_\ell)\}_{\ell=1}^N$ of size*

$$N = \log(n/\delta) 2^{\mathcal{O}(\log(1/\epsilon_3) + \mathrm{polylog}(1/\epsilon_1))}, \tag{S10}$$

where x_ℓ is sampled from $\mathcal{D}$ and y_ℓ is an estimator of $\mathrm{Tr}(O\rho(x_\ell))$ such that $|y_\ell - \mathrm{Tr}(O\rho(x_\ell))| \leq \epsilon_2$, the ML algorithm can produce $h^*(x)$ that achieves prediction error

$$\mathop{\mathbb{E}}_{x\sim\mathcal{D}} |h^*(x) - \mathrm{Tr}(O\rho(x))|^2 \leq (\epsilon_1 + \epsilon_2)^2 + \epsilon_3 \tag{S11}$$

with probability at least $1-\delta$. The training time for constructing the hypothesis function h and the prediction time for computing $h^*(x)$ are upper bounded by $\mathcal{O}(nN) = n\log(n/\delta)2^{\mathcal{O}(\log(1/\epsilon_3)+\mathrm{polylog}(1/\epsilon_1))}$.

In the ML problem formulated in the main text and Section III A, the training data $\{x_\ell, y_\ell\}_{\ell=1}^N$ corresponds to a fixed and unknown observable O. However, we may be interested in training an ML model that can predict $\mathrm{Tr}(O\rho(x))$ for a wide range of observables O. In this setting, one could consider a classical dataset $\{x_\ell, \sigma_T(\rho(x_\ell))\}_{\ell=1}^N$ generated by performing classical shadow tomography [12–16] on the ground state $\rho(x_\ell)$ for each x_ℓ in $\ell = 1, \ldots, N$. This is achieved by repeatedly performing T randomized Pauli measurements on each state $\rho(x_\ell)$. Using the classical shadow dataset, we can obtain the following corollary for predicting ground state representations.

Corollary 5. *Let $1/e > \epsilon_1, \epsilon_2, \epsilon_3 > 0$ and $\delta > 0$. Given a training data set $\{x_\ell, \sigma_T(\rho(x_\ell))\}_{\ell=1}^N$ of size*

$$N = \log(n/\delta) 2^{\mathcal{O}(\log(1/\epsilon_3) + \mathrm{polylog}(1/\epsilon_1))}, \tag{S12}$$

where x_ℓ is sampled from an unknown distribution $\mathcal{D}$ and $\sigma_T(\rho(x_\ell))$ is the classical shadow representation of the ground state $\rho(x_\ell)$ using T randomized Pauli measurements. For $T = \mathcal{O}(\log(nN/\delta)/\epsilon_2^2) = \tilde{\mathcal{O}}(\log(n/\delta)/\epsilon_2^2)$, the proposed ML algorithm can learn a ground state representation $\hat{\rho}_{N,T}(x)$ that achieves

$$\mathop{\mathbb{E}}_{x\sim\mathcal{D}} |\mathrm{Tr}(O\hat{\rho}_{N,T}(x)) - \mathrm{Tr}(O\rho(x))|^2 \leq (\epsilon_1 + \epsilon_2)^2 + \epsilon_3 \tag{S13}$$

for any observable O with eigenvalues between -1 and 1 that can be written as a sum of geometrically local observables with probability at least $1-\delta$.

Proof. For any observable O with eigenvalues between -1 and 1 that can be written as a sum of geometrically local observables, we have $O = \sum_{P\in S^{(\mathrm{geo})}} \alpha_P P$, where $S^{(\mathrm{geo})}$ is the set of all geometrically local Pauli observables. From Corollary 4, we have

$$\sum_{P\in S^{(\mathrm{geo})}} |\alpha_P| \leq C \tag{S14}$$

for a constant C. We are going to use the constant C to set the training data size N and the number of randomized measurements T. In particular, we are going to consider

$$N = \log\Big(n/(\delta/(2|S^{(\mathrm{geo})}|))\Big) 2^{\mathcal{O}\left(\log\left(C^2/\epsilon_3\right) + \mathrm{polylog}(C/\epsilon_1)\right)}, \tag{S15}$$

$$T = \mathcal{O}\left(\log\Big(nN/(\delta/(2|S^{(\mathrm{geo})}|))\Big)/(\epsilon_2/C)^2\right). \tag{S16}$$

For any geometrically local Pauli observables $P \in S^{(\mathrm{geo})}$, we can use the classical shadow dataset $\{x_\ell, \sigma_T(\rho(x_\ell))\}_{\ell=1}^N$ to estimate the expectation value of P in the ground states $\rho(x_\ell)$ for all ℓ. This creates a dataset $\{x_\ell, y_\ell^{(P)}\}_{\ell=1}^N$. Under the specified T, Lemma 1 in [1] guarantees that

$$\left| y_\ell^{(P)} - \mathrm{Tr}(P\rho(x_\ell)) \right| < \frac{\epsilon_2}{C}, \tag{S17}$$

for all $\ell = 1, \ldots, N$ and $P \in S^{(\mathrm{geo})}$ with probability at least $1-(\delta/2)$. For each $P \in S^{(\mathrm{geo})}$, we consider $h_P^*(x)$ to be the function produced from Theorem 5. From Theorem 5, we have

$$\mathop{\mathbb{E}}_{x\sim\mathcal{D}} |h_P^*(x) - \mathrm{Tr}(P\rho(x))|^2 \leq \frac{1}{C^2}\left[(\epsilon_1 + \epsilon_2)^2 + \epsilon_3\right] \tag{S18}$$

for all $P \in S^{(\mathrm{geo})}$ with probability at least $1-(\delta/2)$ conditioned on the event given in Eq. (S17) occurs. Using the union bound to combine the two events considered in Eq. (S17) and Eq. (S18), we can ensure that Eq. (S18) holds with probability at least $1-\delta$.

We define the ground state representation produced by the ML algorithm to be

$$\hat{\rho}_{N,T}(x) \triangleq \sum_{P \in S^{(\mathrm{geo})}} h^*_P(x) \left(\frac{P}{2^n}\right). \tag{S19}$$

For the observable O, we have

$$|\operatorname{Tr}(O\hat{\rho}_{N,T}(x)) - \operatorname{Tr}(O\rho(x))| \leq \sum_{P \in S^{(\mathrm{geo})}} |\alpha_P||h^*_P(x) - \operatorname{Tr}(P\rho(x))|. \tag{S20}$$

By the Cauchy-Schwarz inequality, Eq. (S14), and Eq. (S18), we have

$$\mathop{\mathbb{E}}_{x\sim\mathcal{D}} |\operatorname{Tr}(O\hat{\rho}_{N,T}(x)) - \operatorname{Tr}(O\rho(x))|^2 \tag{S21}$$

$$\leq \sum_{P_1,P_2 \in S^{(\mathrm{geo})}} |\alpha_{P_1}||\alpha_{P_2}| \mathop{\mathbb{E}}_{x\sim\mathcal{D}} |h^*_{P_1}(x) - \operatorname{Tr}(P_1\rho(x))||h^*_{P_2}(x) - \operatorname{Tr}(P_2\rho(x))| \tag{S22}$$

$$\leq \sum_{P_1,P_2 \in S^{(\mathrm{geo})}} |\alpha_{P_1}||\alpha_{P_2}| \sqrt{\mathop{\mathbb{E}}_{x\sim\mathcal{D}} |h^*_{P_1}(x) - \operatorname{Tr}(P_1\rho(x))|^2} \sqrt{\mathop{\mathbb{E}}_{x\sim\mathcal{D}} |h^*_{P_2}(x) - \operatorname{Tr}(P_2\rho(x))|^2} \tag{S23}$$

$$\leq \left(\sum_{P \in S^{(\mathrm{geo})}} |\alpha_P|\right)^2 \frac{1}{C^2}\left[(\epsilon_1+\epsilon_2)^2 + \epsilon_3\right] \leq (\epsilon_1+\epsilon_2)^2 + \epsilon_3. \tag{S24}$$

This concludes the proof of the corollary. □

C. ℓ_1-Norm bound on coefficients of linear hypothesis

We now justify our choice of the hyperparameter $B > 0$ such that $\|\mathbf{w}\|_1 \leq B$. From Section I, we constructed a function that approximates the ground state property. Explicitly, this function is defined as

$$g(x) \triangleq \sum_{P \in S^{(\mathrm{geo})}} \sum_{x' \in X_P} f_P(x') \mathbb{1}[x \in T_{x',P}] = \mathbf{w}' \cdot \phi(x), \tag{S25}$$

where in this case, the vector of coefficients $\mathbf{w}'$, indexed by $x' \in X_P, P \in S^{(\mathrm{geo})}$, is defined as

$$\mathbf{w}'_{x',P} \triangleq f_P(x'). \tag{S26}$$

Thus, we see that the ML model, which learns functions of this form, has the capacity to approximate the target ground state property $\operatorname{Tr}(O\rho(x))$. The actual function we learn, $h^*(x) = \mathbf{w}^* \cdot \phi(x)$ could differ significantly from $g(x) = \mathbf{w}' \cdot \phi(x)$ because $\mathbf{w}'$ is unknown. Nevertheless, we can utilize an upper bound $\|\mathbf{w}'\|_1 \leq B$ to restrict the hypothesis set of the ML algorithm to functions of the form $h(x) = \mathbf{w} \cdot \phi(x)$ such that $\|\mathbf{w}\|_1 \leq B$. Thus, we find an upper bound on $\|\mathbf{w}'\|_1$ in the following lemma.

Lemma 14 (ℓ_1-Norm bound)**.** *Let $\mathbf{w}'$ be the vector of coefficients defined in Eq. (S26). Then, we have the following bound on $\|\mathbf{w}'\|_1$:*

$$\|\mathbf{w}'\|_1 = \sum_{P \in S^{(\mathrm{geo})}} \sum_{x' \in X_P} |f_P(x')| = 2^{\mathcal{O}(\mathrm{polylog}(1/\epsilon_1))}. \tag{S27}$$

Proof. First, we can analyze the $|f_P(x')|$ term. Recall that f_P is just $\alpha_P \operatorname{Tr}(P\rho(\chi_P(x)))$, where $\chi_P(x) \in [-1,1]^m$ sets parameters outside of I_P to 0. Thus, we can bound its absolute value by

$$|f_P(x)| = |\alpha_P||\operatorname{Tr}(P\rho(\chi_P(x)))| \leq |\alpha_P|. \tag{S28}$$

Plugging this into the ℓ_1-norm of $\mathbf{w}'$, we have

$$\|\mathbf{w}'\|_1 = \sum_{P \in S^{(\mathrm{geo})}} \sum_{x' \in X_P} |f_P(x')| \tag{S29a}$$

$$\leq \sum_{P \in S^{(\text{geo})}} |X_P||f_P(x')| \tag{S29b}$$

$$\leq \max_{P \in S^{(\text{geo})}} |X_P| \sum_{Q \in S^{(\text{geo})}} |\alpha_Q|. \tag{S29c}$$

Thus, it suffices to count the number of elements in X_P. Recall in Definition 3 that X_P is defined such that the parameter values for $c' \notin I_P$ are fixed to 0 while for $c' \in I_P$, $x_{c'}$ can be any value in $\{0, \pm\delta_2, \pm 2\delta_2, \ldots, \pm 1\}$. Hence, it is clear that

$$|X_P| \leq |\{0, \pm\delta_2, \pm 2\delta_2, \ldots, \pm 1\}|^{|I_P|} \leq \left(\frac{2}{\delta_2} + 1\right)^{|I_P|}. \tag{S30}$$

Moreover, by our choice of δ_2 in Eq. (S3), we have

$$|X_P| \leq \left(2\left\lceil \frac{2\sqrt{C'|I_P|}}{\epsilon_1} \right\rceil + 1\right)^{|I_P|}. \tag{S31}$$

Now, it remains to bound the size of I_P, defined in Eq. (S11). This size is simply the number of parameters that h_j depends on for some h_j with $d_{\text{obs}}(h_j, P) \leq \delta_1$. By Eq. (S5), we can bound the number of such h_j:

$$\sum_{j : d_{\text{obs}}(h_j, P) \leq \delta_1} 1 \leq b_d + c_d \delta_1^d. \tag{S32}$$

Moreover, we assume that each h_j depends on $\mathcal{O}(1)$ parameters. Suppose that each h_j depends on at most q parameters. Then, we can bound the size of I_P by

$$|I_P| \leq q(b_d + c_d \delta_1^d). \tag{S33}$$

Utilizing this bound in Eq. (S31), we obtain

$$|X_P| \leq \left(2\left\lceil \frac{2\sqrt{C'q(b_d + c_d\delta_1^d)}}{\epsilon_1} \right\rceil + 1\right)^{q(b_d + c_d\delta_1^d)}. \tag{S34}$$

Plugging this into our ℓ_1-norm bound from Eq. (S29c), we have

$$\|\mathbf{w}'\|_1 = \sum_{P \in S^{(\text{geo})}} \sum_{x' \in X_P} |f_P(x')| \tag{S35a}$$

$$\leq \left(2\left\lceil \frac{2\sqrt{C'q(b_d + c_d\delta_1^d)}}{\epsilon_1} \right\rceil + 1\right)^{q(b_d + c_d\delta_1^d)} \sum_{Q \in S^{(\text{geo})}} |\alpha_Q| \tag{S35b}$$

$$\leq D\left(2\left\lceil \frac{2\sqrt{C'q(b_d + c_d\delta_1^d)}}{\epsilon_1} \right\rceil + 1\right)^{q(b_d + c_d\delta_1^d)}, \tag{S35c}$$

where the second inequality follows from Corollary 4, taking D as this constant. We can simplify this expression further by using that $\delta_1 = C_{\max} \log^2(2C/\epsilon_1)$ for sufficiently small ϵ_1 according to Eq. (S1).

$$\|\mathbf{w}'\|_1 = \sum_{P \in S^{(\text{geo})}} \sum_{x' \in X_P} |f_P(x')| \tag{S36a}$$

$$\leq D\left(2\left\lceil \frac{2\sqrt{C'q(b_d + c_d(C_{\max}\log^2(2C/\epsilon_1))^d)}}{\epsilon_1} \right\rceil + 1\right)^{q(b_d + c_d(C_{\max}\log^2(2C/\epsilon_1))^d)} \tag{S36b}$$

$$= \left(\frac{\log^{2d}(1/\epsilon_1)}{\epsilon_1}\right)^{\mathcal{O}(\log^{2d}(1/\epsilon_1))} \tag{S36c}$$

$$= \left(\frac{1}{\epsilon_1}\right)^{\mathcal{O}(\log^{2d}(1/\epsilon_1))} \left(\log^{2d}(1/\epsilon_1)\right)^{\mathcal{O}(\log^{2d}(1/\epsilon_1))} \tag{S36d}$$

$$= 2^{\mathcal{O}(\log^{2d+1}(1/\epsilon_1))} \tag{S36e}$$

$$= 2^{\mathcal{O}(\text{polylog}(1/\epsilon_1))}, \tag{S36f}$$

which is the promised scaling. □

D. Training error bound

Using the results in Section I C, we can derive a bound on the training error of $g(x) = \mathbf{w}' \cdot \phi(x)$ discussed in the previous section. The existence of $\mathbf{w}'$ then guarantees that the function $h^*(x) = \mathbf{w}^* \cdot \phi(x)$ found by performing optimization to minimize training error will also yield a training error close to zero. To prove this rigorously, we first write a precise definition of training error.

Definition 8 (Training error)**.** *Given a function $h(x)$ and a training dataset $\{(x_\ell, y_\ell)\}_{\ell=1}^N$. The* training error *is defined as*

$$\hat{R}(h) = \min_{\mathbf{w}} \frac{1}{N} \sum_{\ell=1}^{N} |h(x_\ell) - y_\ell|^2. \tag{S37}$$

We can bound the training error in the following lemma.

Lemma 15 (Detailed restatement of Lemma 1)**.** *The function*

$$g(x) = \sum_{P \in S^{(\mathrm{geo})}} \sum_{x' \in X_P} f_P(x') \mathbb{1}[x \in T_{x',P}] = \mathbf{w}' \cdot \phi(x), \tag{S38}$$

achieves training error

$$\hat{R}(g) \leq (\epsilon_1 + \epsilon_2)^2, \tag{S39}$$

where the training error is defined in Definition 8.

Proof. This lemma follows directly from Theorem 3. Let ℓ^* be defined as

$$\ell^* = \underset{1 \leq \ell \leq N}{\operatorname{argmax}} |g(x_\ell) - y_\ell|^2. \tag{S40}$$

Then, the training error can be bounded above by

$$\hat{R}(g) \leq |g(x_{\ell^*}) - y_{\ell^*}|^2 \leq \left(|g(x_{\ell^*}) - \mathrm{Tr}(O\rho(x_{\ell^*}))| + |\,\mathrm{Tr}(O\rho(x_{\ell^*})) - y_{\ell^*}|\right)^2, \tag{S41}$$

where the last inequality follows by triangle inequality. Here, the second term can be bounded by ϵ_2 using definition of our training labels y_ℓ. For the first term, let D be a constant such that

$$\sum_{P \in S^{(\mathrm{geo})}} |\alpha_P| \leq D, \tag{S42}$$

using Corollary 4. Then, by Theorem 3, we have

$$|g(x_{\ell^*}) - \mathrm{Tr}(O\rho(x_{\ell^*}))| \leq \epsilon_1 + \epsilon_2. \tag{S43}$$

Putting everything together, we have

$$\hat{R}(g) \leq (\epsilon_1 + \epsilon_2)^2, \tag{S44}$$

which is the claimed result. □

Now consider the function $h^*(x) = \mathbf{w}^* \cdot \phi(x)$, where $\mathbf{w}^*$ is obtained by minimizing the training error, such that the training error is larger than the minimum training error by at most $\epsilon_3/2$. We can achieve this using an optimization algorithm described in Section III F. Formally, we have the following inequality,

$$\hat{R}(h^*) \leq \frac{\epsilon_3}{2} + \min_{\substack{\mathbf{w} \in \mathbb{R}^{m_\phi} \\ \|\mathbf{w}\|_1 \leq B}} \frac{1}{N} \sum_{\ell=1}^{N} |\mathbf{w} \cdot \phi(x_\ell) - \mathrm{Tr}(O\rho(x_\ell))|^2. \tag{S45}$$

Because we have set $B = 2^{\mathrm{polylog}(1/\epsilon_1)}$,

$$\|\mathbf{w}'\|_1 = \sum_{P \in S^{(\mathrm{geo})}} \sum_{x' \in X_P} |f_P(x')| \leq 2^{\mathcal{O}(\mathrm{polylog}(1/\epsilon_1))} = B. \tag{S46}$$

Therefore, the minimum training error must be at most $\hat{R}(g)$,

$$\min_{\substack{\mathbf{w} \in \mathbb{R}^{m_\phi} \\ \|\mathbf{w}\|_1 \leq B}} \frac{1}{N} \sum_{\ell=1}^{N} |\mathbf{w} \cdot \phi(x_\ell) - \mathrm{Tr}(O\rho(x_\ell))|^2 \leq \hat{R}(g). \tag{S47}$$

Together, we have

$$\hat{R}(h^*) \leq \hat{R}(g) + \frac{\epsilon_3}{2} \leq (\epsilon_1 + \epsilon_2)^2 + \frac{\epsilon_3}{2}. \tag{S48}$$

The last inequality follows from Lemma 15.

E. Prediction error bound

With this bound on the training error, it remains to find a bound on the prediction error of our hypothesis function. To this end, we can use a standard result from machine learning theory about the prediction error of ℓ_1-norm-constrained linear hypotheses trained using the LASSO algorithm [9–11].

Theorem 6 (Theorem 11.16 in [11]). *Let $\mathcal{X} \subseteq \mathbb{R}^A$ and $\mathcal{H} = \{\mathbf{x} \in \mathcal{X} \mapsto \mathbf{w} \cdot \mathbf{x} : \|\mathbf{w}\|_1 \leq B\}$. Let $S = ((\mathbf{x}_1, y_1), \ldots, (\mathbf{x}_N, y_N)) \in (\mathcal{X} \times \mathcal{Y})^N$. Let $\mathcal{D}$ denote a distribution over $\mathcal{X} \times \mathcal{Y}$ according to which the training data S is drawn. Assume that there exists $r_\infty > 0$ such that for all $\mathbf{x} \in \mathcal{X}$, $\|\mathbf{x}\|_\infty \leq r_\infty$ and $M > 0$ such that $|h(x) - y| \leq M$ for all $(x, y) \in \mathcal{X} \times \mathcal{Y}$. Then, for any $\delta > 0$, with probability at least $1 - \delta$, each of the following inequalities holds for all $h \in \mathcal{H}$:*

$$\mathop{\mathbb{E}}_{(x,y)\sim\mathcal{D}} |h(x) - y|^2 \triangleq R(h) \leq \hat{R}_S(h) + 2r_\infty BM\sqrt{\frac{2\log(2A)}{N}} + M^2\sqrt{\frac{\log\frac{1}{\delta}}{2N}} \tag{S49}$$

where $R(h)$ is the prediction error for the hypothesis h and $\hat{R}_S(h)$ is the training error of h on the training data S.

We can use this theorem to prove the prediction error bound in Theorem 5.

Proof of prediction error in Theorem 5. We utilize Theorem 6 as well as our established lemmas.

First, we demonstrate that we satisfy the conditions of the theorem in our setting. Here, we view h in Theorem 6 as a function of the higher-dimensional feature vector $\phi(x)$ rather than the m-dimensional vector $x \in [-1, 1]^m$ so that h is a linear hypothesis. In this perspective, our input space $\mathcal{X}$ is the feature space $\{0, 1\}^{m_\phi} \subseteq \mathbb{R}^{m_\phi}$, as the indicator functions we are evaluating only take 0-1 values. In our case, the dimension A is given by

$$A = m_\phi \triangleq \sum_{P \in S^{(\mathrm{geo})}} |X_P|. \tag{S50}$$

Moreover, the training data we are given is $S = ((\phi(x_1), y_1), \ldots, (\phi(x_N), y_N)) \in (\mathcal{X} \times \mathcal{Y})^N$, where y_ℓ is such that

$$|y_\ell - \mathrm{Tr}(O\rho(x_\ell))| \leq \epsilon_2. \tag{S51}$$

Again, we are thinking of h as a function that takes the input $\phi(x)$. Furthermore, since $\phi(x_\ell) \in \{0, 1\}^{m_\phi}$ for all $\ell = 1, \ldots, N$, we can see that $\|\phi(x_\ell)\|_\infty \leq 1 = r_\infty$. Moreover, the hypothesis class $\mathcal{H}$ is given by the set of the functions of the same form as h, i.e., $\mathcal{H} = \{\phi(x) \in \mathcal{X} \mapsto \mathbf{w} \cdot \phi(x) : \|\mathbf{w}\|_1 \leq B\}$ with $B = 2^{\mathrm{polylog}(1/\epsilon)}$. By considering $M = 2^{\mathcal{O}(\mathrm{polylog}(1/\epsilon_1))}$, we also have $|h(x_\ell) - y_\ell| \leq M$ for all $\ell = 1, \ldots, N$ because

$$|h(x_\ell) - y_\ell| \leq |\mathbf{w} \cdot \phi(x_\ell)| + |y_\ell| \leq \|\mathbf{w}\|_1 \|\phi(x)\|_\infty + 2 \leq 2^{\mathcal{O}(\mathrm{polylog}(1/\epsilon_1))} + 2 = 2^{\mathcal{O}(\mathrm{polylog}(1/\epsilon_1))}, \tag{S52}$$

where the second inequality follows by Hölder's inequality. Furthermore, by Eq. (S48), the learned model $h^*(x) = \mathbf{w}^* \cdot \phi(x)$ achieves $\hat{R}(h^*) \leq (\epsilon_1 + \epsilon_2)^2 + (\epsilon_3/2)$. Thus, by Theorem 6,

$$R(h^*) \leq (\epsilon_1 + \epsilon_2)^2 + \frac{\epsilon_3}{2} + 2BM\sqrt{\frac{2\log(2m_\phi)}{N}} + M^2\sqrt{\frac{\log\frac{1}{\delta}}{2N}} \tag{S53}$$

with probability at least $1 - \delta$. In order to bound the prediction error above by $(\epsilon_1 + \epsilon_2)^2 + \epsilon_3$, we need N to be large enough such that

$$2BM\sqrt{\frac{2\log(2m_\phi)}{N}} + M^2\sqrt{\frac{\log\frac{1}{\delta}}{2N}} \leq \frac{\epsilon_3}{2}. \tag{S54}$$

We can upper bound m_ϕ using the same approach as in the proof of Lemma 14. Explicitly, using Eq. (S34) and Eq. (S36), we have

$$m_\phi = \sum_{P \in S^{(\mathrm{geo})}} |X_P| \leq \sum_{P \in S^{(\mathrm{geo})}} \left(2\left\lceil \frac{\sqrt{C' q(b_d + c_d \delta_1^d)}}{\epsilon_1} \right\rceil + 1\right)^{q(b_d + c_d\delta_1^d)} \tag{S55a}$$

$$= 2^{\mathcal{O}(\mathrm{polylog}(1/\epsilon_1))}\mathcal{O}(n), \tag{S55b}$$

where the last equality follows because $|S^{(\mathrm{geo})}| = \mathcal{O}(n)$. Plugging everything into the left hand side of Eq. (S54), we have

$$2BM\sqrt{\frac{2\log(2m_\phi)}{N}} + M^2\sqrt{\frac{\log\frac{1}{\delta}}{2N}} \tag{S56a}$$

$$\leq 2\sqrt{2}\left(2^{\mathcal{O}(\mathrm{polylog}(1/\epsilon_1))}\right)^2\sqrt{\frac{\log\left(2\cdot 2^{\mathcal{O}(\mathrm{polylog}(1/\epsilon_1))}\mathcal{O}(n)\right)}{N}} \tag{S56b}$$

$$+\frac{1}{\sqrt{2}}\left(2^{\mathcal{O}(\mathrm{polylog}(1/\epsilon_1))}\right)^2\sqrt{\frac{\log\frac{1}{\delta}}{N}} \tag{S56c}$$

$$= 2^{\mathcal{O}(\mathrm{polylog}(1/\epsilon_1))}\frac{1}{\sqrt{N}}\left(\sqrt{\mathcal{O}(\mathrm{polylog}(1/\epsilon_1)) + \mathcal{O}(\log(n))} + \sqrt{\log\frac{1}{\delta}}\right). \tag{S56d}$$

To upper bounded the above by $\frac{\epsilon_3}{2}$, we choose

$$N = \frac{4}{\epsilon_3^2}\left(2^{\mathcal{O}(\mathrm{polylog}(1/\epsilon_1))}\right)^2\left(\sqrt{\mathcal{O}(\mathrm{polylog}(1/\epsilon_1)) + \mathcal{O}(\log(n))} + \sqrt{\log\frac{1}{\delta}}\right)^2 \tag{S57a}$$

$$= 2^{\mathcal{O}(\log(1/\epsilon_3)+\mathrm{polylog}(1/\epsilon_1))}\log(n/\delta). \tag{S57b}$$

Together, the training data size N given above guarantees that $R(h^*) \leq (\epsilon_1+\epsilon_2)^2 + \epsilon_3$ with probability at least $1-\delta$. □

F. Computational time for training and prediction

Finally, we find the computation time required for the ML algorithm's training and prediction. To this end, we utilize standard results about the training time of the LASSO algorithm [17].

Proof of computational time in Theorem 5. The training time is dominated by the time required for ℓ_1-regularized regression (LASSO) over the feature space defined by the feature map ϕ. It is well-known that to obtain a training error at most $(\epsilon_3/2)$ larger than the optimal function value, the LASSO algorithm on the feature space can be executed in time $\mathcal{O}\left(\frac{m_\phi \log m_\phi}{\epsilon_3^2}\right)$ [17], where m_ϕ is the dimension of the feature space. By Eq. (S55b), we know that

$$m_\phi = \mathcal{O}(n)2^{\mathcal{O}(\mathrm{polylog}(1/\epsilon_1))} \tag{S58}$$

Plugging this into the time required for LASSO, we have

$$\mathcal{O}\left(\frac{m_\phi\log m_\phi}{\epsilon_3^2}\right) = \mathcal{O}\left(\frac{\mathcal{O}(n)2^{\mathcal{O}(\mathrm{polylog}(1/\epsilon_1))}\log\left(\mathcal{O}(n)2^{\mathcal{O}(\mathrm{polylog}(1/\epsilon_1))}\right)}{\epsilon_3^2}\right) \tag{S59a}$$

$$= \mathcal{O}(n2^{\mathcal{O}(\log(1/\epsilon_3)+\mathrm{polylog}(1/\epsilon_1))}(\mathcal{O}(\mathrm{polylog}(1/\epsilon_1)) + \mathcal{O}(\log(n)))) \tag{S59b}$$

$$= n\log n\, 2^{\mathcal{O}(\log(1/\epsilon_3)+\mathrm{polylog}(1/\epsilon_1))} \tag{S59c}$$

$$= \mathcal{O}(nN), \tag{S59d}$$

where the last equality follows by the definition of the training data size N.

The prediction time is the amount of time it takes to compute $h^*(x) = \mathbf{w}^*\cdot\phi(x_\ell)$, which takes time $\mathcal{O}(m_\phi)$. This can also be upper bounded by $\mathcal{O}(nN)$. □

IV. DETAILS OF NUMERICAL EXPERIMENTS

For the numerical experiments, we consider the two-dimensional antiferromagnetic Heisenberg model. In this setting, spin-1/2 particles are placed on sites in a 2D lattice. The Hamiltonian is

$$H = \sum_{\langle ij\rangle} J_{ij}(X_iX_j + Y_iY_j + Z_iZ_j), \tag{S1}$$

where the summation ranges over all pairs $\langle ij\rangle$ of neighboring sites on the lattice and the couplings $\{J_{ij}\}$ are sampled uniformly from the interval $[0,2]$. Here, the parameter x is a list of all couplings J_{ij} so that the dimension of the parameter space is $m = O(n)$, where n is the system size. This is the same class of Hamiltonians considered in previous work [1]. We are interested in predicting ground state properties, which in this case are the two-body correlation functions for each pair of qubits on the lattice. In particular, this correlation function is the expectation value of

$$C_{ij} = \frac{1}{3}(X_iX_j + Y_iY_j + Z_iZ_j), \tag{S2}$$

for each pair of qubits $\langle ij\rangle$.

We generated training and testing data for this model using the same method as [1]. For completeness, we briefly discuss this here. For each parameter vector of random couplings sampled uniformly from $[0,2]$, we approximated the ground state using the density-matrix renormalization group (DMRG) [18] based on matrix product states (MPS) [19]. We first consider an initial random MPS with bond dimension $\chi = 10$ and variationally optimize it using a singular value decomposition cutoff of 10^{-8}. We terminate the DMRG runs when the change in energy is less than 10^{-4}. After DMRG converges, we perform randomized Pauli measurements by locally rotating into the corresponding Pauli bases and sampling the rotated state [20]. In this work, we utilize two different data sets: one which is the same as in [1] and the other which is generated in the same way but contains more data points.

We consider classical machine learning models given by first performing a feature mapping ϕ on the input vector x and then running ℓ_1-regularized regression (LASSO) over the feature $\phi(x)$ space, as described in Section III A. However, while the indicator function feature map was a useful tool to obtain our rigorous guarantees, it is often hard to discretize a high-dimension parameter space into X_P in practice. Thus, we instead utilize random Fourier features [21]. One can think of this as a single layer of a randomly initialized neural network. Explicitly, this feature map is

$$\phi : z \mapsto \begin{pmatrix} \cos\left(\frac{\gamma}{\sqrt{l}}(\omega_1 \cdot z)\right) \\ \sin\left(\frac{\gamma}{\sqrt{l}}(\omega_1 \cdot z)\right) \\ \vdots \\ \cos\left(\frac{\gamma}{\sqrt{l}}(\omega_R \cdot z)\right) \\ \sin\left(\frac{\gamma}{\sqrt{l}}(\omega_R \cdot z)\right) \end{pmatrix}, \tag{S3}$$

where l is the length of the vector z, $\gamma > 0$ and $R > 0$ are tunable hyperparameters, and ω_i are l-dimensional vectors sampled from a multivariate standard normal distribution. Here, for each vector z, $\phi(z)$ is a $2R$-dimensional vector. Thus, the hyperparameter R determines the length of the feature vector. We consider a set of different hyperparameters:

$$R \in \{5, 10, 20, 40\}, \tag{S4}$$
$$\gamma \in \{0.4, 0.5, 0.6, 0.65, 0.7, 0.75\}. \tag{S5}$$

Using this feature map, the ML algorithm is implemented as follows. First, we decompose x into several vectors corresponding to local regions of a given local term of the Hamiltonian. This is analogous to the discretization of the parameter space using X_P. Explicitly, the decomposition is performed in the following way. First, recall that in the 2D antiferromagnetic Heisenberg model, qubits are placed on sites in a 2D lattice. Thus, each local term can be viewed as an edge between neighboring sites on the lattice. We construct a local region around this edge by including all edges within an ℓ_1-distance δ_1. This is analogous to Eq. (S11). Now, for each vector resulting from the decomposition of x, we apply the feature map ϕ and concatenate all vectors together to obtain $\phi(x)$. Finally, we run the LASSO algorithm using `scikit-learn`, a Python package [22]. Here, LASSO optimizes the objective function

$$\frac{1}{2N}\|y - Xw\|_2^2 + \alpha\|w\|_1, \tag{S6}$$

where N is the amount of training data, y is a vector of the training data labels $\{y_\ell\}_{\ell=1}^N$, X is a matrix of the training data inputs $\{x_\ell\}_{\ell=1}^N$, w is a vector of coefficients we want to learn, and $\alpha > 0$ is a regularization parameter. We consider a set of different regularization parameters

$$\alpha \in \{2^{-8}, 2^{-7}, 2^{-6}, 2^{-5}\}. \tag{S7}$$

We consider several different classical ML models, corresponding to these choices of hyperparameters R, γ, α. Thus, we perform model selection to determine the optimal choice of these hyperparameters. To this end, we consider M different values of the parameter $x = \{J_{ij}\}$, where M is as big as 900. From these M data points, we randomly choose half of these points as training data (i.e., $N = M/2$) and the remaining half is test data. For each ground state property we want to predict, we choose one value of each of R, γ, α such that the root-mean-square error is minimized when performing 4-fold cross-validation, which is also implemented using `scikit-learn`. Finally, we test the performance of the ML model with these chosen hyperparameters using the test data.

For each vector x that we tested on, we would predict the correlation functions for all pairs of qubits $\langle ij \rangle$. Hence, the prediction error is averaged over a large number of predictions. Despite $M/2$ being only of around 50, the prediction errors reported in the plots are statistically sound given the large total number of predictions. The standard deviation of the exact correlation functions in the data varies slightly across different system sizes. The standard deviation for system size 4×5 is around 0.192, 5×5 is around 0.199, 6×5 is around 0.187, 7×5 is around 0.193, 8×5 is around 0.190, 9×5 is around 0.187. When the standard deviation is smaller, the prediction error will also be smaller. To judge the difficulty to predict the correlation functions across different system sizes, we normalize the standard deviation to be the average standard deviation of 0.191. We also include experiments where we vary the training data size N or the classical shadow size T, i.e., the number of randomized Pauli measurements used to approximate the ground state. The left figure of Figure 2A fixes the training set size N to be 50, system size n to be $9 \times 5 = 45$, and varies the classical shadow size $T \in \{50, 100, 250, 500, 1000\}$. The center figure of Figure 2A fixes the shadow size T to be 500, system size n to be $9 \times 5 = 45$, and varies the training data size varies from $N \in \{10, 30, 50, 75, 90, 150, 250, 350, 450\}$. The right figure of Figure 2A fixes the training set size $N = 250$ and shadow size $T = 500$, and varies the system size from 4×5 to 9×5. The numerical results of these experiments are summarized in Figure 2.

---

[1] Hsin-Yuan Huang, Richard Kueng, Giacomo Torlai, Victor V Albert, and John Preskill. Provably efficient machine learning for quantum many-body problems. *Science*, 377(6613):eabk3333, 2022.

[2] Sven Bachmann, Spyridon Michalakis, Bruno Nachtergaele, and Robert Sims. Automorphic equivalence within gapped phases of quantum lattice systems. *Commun. Math. Phys.*, 309(3):835–871, 2012.

[3] Elliott H Lieb and Derek W Robinson. The finite group velocity of quantum spin systems. In *Statistical mechanics*, pages 425–431. Springer, 1972.

[4] Matthew B Hastings and Xiao-Gang Wen. Quasiadiabatic continuation of quantum states: The stability of topological ground-state degeneracy and emergent gauge invariance. *Phys. Rev. B*, 72(4):045141, 2005.

[5] Tobias J Osborne. Simulating adiabatic evolution of gapped spin systems. *Phys. Rev. A*, 75(3):032321, 2007.

[6] Matthew B Hastings. Locality in quantum systems. *arXiv:1008.5137*, 2010.

[7] Sergey Bravyi, Matthew B Hastings, and Frank Verstraete. Lieb-robinson bounds and the generation of correlations and topological quantum order. *Phys. Rev. Lett.*, 97(5):050401, 2006.

[8] Iosif Pinelis. Exact lower and upper bounds on the incomplete gamma function. *arXiv preprint arXiv:2005.06384*, 2020.

[9] Fadil Santosa and William W. Symes. Linear inversion of band-limited reflection seismograms. *SIAM Journal on Scientific and Statistical Computing*, 7(4):1307–1330, 1986.

[10] Robert Tibshirani. Regression shrinkage and selection via the lasso. *Journal of the Royal Statistical Society: Series B (Methodological)*, 58(1):267–288, 1996.

[11] Mehryar Mohri, Afshin Rostamizadeh, and Ameet Talwalkar. *Foundations of machine learning.* The MIT Press, 2018.

[12] Hsin-Yuan Huang, Richard Kueng, and John Preskill. Predicting many properties of a quantum system from very few measurements. *Nat. Phys.*, 16:1050—1057, 2020.

[13] Andreas Elben, Richard Kueng, Hsin-Yuan Huang, Rick van Bijnen, Christian Kokail, Marcello Dalmonte, Pasquale Calabrese, Barbara Kraus, John Preskill, Peter Zoller, and Benoît Vermersch. Mixed-state entanglement from local randomized measurements. *Phys. Rev. Lett.*, 125:200501, 2020.

[14] Andreas Elben, Steven T Flammia, Hsin-Yuan Huang, Richard Kueng, John Preskill, Benoît Vermersch, and Peter Zoller. The randomized measurement toolbox. *arXiv preprint arXiv:2203.11374*, 2022.

[15] Kianna Wan, William J Huggins, Joonho Lee, and Ryan Babbush. Matchgate shadows for fermionic quantum simulation. *arXiv preprint arXiv:2207.13723*, 2022.

[16] Kaifeng Bu, Dax Enshan Koh, Roy J Garcia, and Arthur Jaffe. Classical shadows with pauli-invariant unitary ensembles. *arXiv preprint arXiv:2202.03272*, 2022.

[17] Elad Hazan and Tomer Koren. Linear regression with limited observation. *arXiv preprint arXiv:1206.4678*, 2012.

[18] Steven R White. Density matrix formulation for quantum renormalization groups. *Physical review letters*,

69(19):2863, 1992.

[19] Ulrich Schollwoeck. The density-matrix renormalization group in the age of matrix product states. *Ann. Phys.*, 326(1):96 – 192, 2011. January 2011 Special Issue.

[20] Andrew J. Ferris and Guifre Vidal. Perfect sampling with unitary tensor networks. *Phys. Rev. B*, 85:165146, 2012.

[21] Ali Rahimi and Benjamin Recht. Random features for large-scale kernel machines. *Advances in neural information processing systems*, 20, 2007.

[22] F. Pedregosa, G. Varoquaux, A. Gramfort, V. Michel, B. Thirion, O. Grisel, M. Blondel, P. Prettenhofer, R. Weiss, V. Dubourg, J. Vanderplas, A. Passos, D. Cournapeau, M. Brucher, M. Perrot, and E. Duchesnay. Scikit-learn: Machine learning in Python. *J. Mach. Learn. Res.*, 12:2825–2830, 2011.
